# YB-1 unwinds mRNA secondary structures *in vitro* and negatively regulates stress granule assembly in HeLa cells

**DOI:** 10.1093/nar/gkab748

**Published:** 2021-09-01

**Authors:** Karina Budkina, Krystel El Hage, Marie-Jeanne Clément, Bénédicte Desforges, Ahmed Bouhss, Vandana Joshi, Alexandre Maucuer, Loic Hamon, Lev P Ovchinnikov, Dmitry N Lyabin, David Pastré

**Affiliations:** SABNP, Univ Evry, INSERM U1204, Université Paris-Saclay, 91025 Evry, France; Institute of Protein Research, Russian Academy of Sciences, Pushchino, 142290, Russian Federation; SABNP, Univ Evry, INSERM U1204, Université Paris-Saclay, 91025 Evry, France; SABNP, Univ Evry, INSERM U1204, Université Paris-Saclay, 91025 Evry, France; SABNP, Univ Evry, INSERM U1204, Université Paris-Saclay, 91025 Evry, France; SABNP, Univ Evry, INSERM U1204, Université Paris-Saclay, 91025 Evry, France; SABNP, Univ Evry, INSERM U1204, Université Paris-Saclay, 91025 Evry, France; SABNP, Univ Evry, INSERM U1204, Université Paris-Saclay, 91025 Evry, France; SABNP, Univ Evry, INSERM U1204, Université Paris-Saclay, 91025 Evry, France; Institute of Protein Research, Russian Academy of Sciences, Pushchino, 142290, Russian Federation; Institute of Protein Research, Russian Academy of Sciences, Pushchino, 142290, Russian Federation; SABNP, Univ Evry, INSERM U1204, Université Paris-Saclay, 91025 Evry, France

## Abstract

In the absence of the scanning ribosomes that unwind mRNA coding sequences and 5′UTRs, mRNAs are likely to form secondary structures and intermolecular bridges. Intermolecular base pairing of non polysomal mRNAs is involved in stress granule (SG) assembly when the pool of mRNAs freed from ribosomes increases during cellular stress. Here, we unravel the structural mechanisms by which a major partner of dormant mRNAs, YB-1 (YBX1), unwinds mRNA secondary structures without ATP consumption by using its conserved cold-shock domain to destabilize RNA stem/loops and its unstructured C-terminal domain to secure RNA unwinding. At endogenous levels, YB-1 facilitates SG disassembly during arsenite stress recovery. In addition, overexpression of wild-type YB-1 and to a lesser extent unwinding-defective mutants inhibit SG assembly in HeLa cells. Through its mRNA-unwinding activity, YB-1 may thus inhibit SG assembly in cancer cells and package dormant mRNA in an unfolded state, thus preparing mRNAs for translation initiation.

## INTRODUCTION

In proliferating cells such as cancer cells, mRNAs are mostly associated with scanning ribosomes to synthesize proteins, and only a small fraction of mRNAs remain dormant (1). However, during many forms of cellular stresses such as oxidative stress, hypoxia, and osmotic stress, specific kinases are activated to block translation initiation, notably by preventing cap-dependent translation (2). As most mRNAs rely on cap-dependent translation in mammalian cells, polysome dissociation after stress induction leads to an increase in non polysomal mRNA in the cytoplasm (stalled translation initiation complexes, mRNPs without ribosomes). The association of non polysomal mRNA with self-adhesive RNA-binding proteins such as G3BP-1 subsequently promotes the assembly of liquid-like mRNA-rich condensates in the cytoplasm called stress granules (SGs) (3–6). In addition to the important contribution of self-adhesive RNA-binding proteins (RBPs), intermolecular RNA–RNA interactions also contribute to SG assembly (7). Indeed, the non polysomal mRNA concentration is higher in SG condensates than in the surrounding cytoplasm. The confinement of mRNAs in SGs then increases the occurrence of intermolecular base pairing between mRNAs. Furthermore, intramolecular base-pairing may direct self-adhesive RBPs in SGs owing to their high affinity for mRNA secondary structures (8). In agreement with the critical role of RNA:RNA interactions in SG assembly, the overexpression of the RNA helicases, eIF4A and DDX19A, was shown to negatively regulate SG assembly (9).

In this study, we considered the putative role of YB-1, an abundant mRNA-binding protein, in SG assembly. YB-1 is a member of the Y-box binding protein family in mammals (10,11). The other members of this family, namely, YB-2 and -3, share a single cold-shock domain (CSD), a high level of identity and, most likely, common functions. Albeit YB-1 is not an ATP-dependent helicase, it forms a linear nucleoprotein filament in the presence of mRNA *in vitro* (12,13), which requires mRNA-unwinding activity. In addition, the single CSD in YB-1 (10,14) has been inherited from cold-shock proteins (CSPs) that originated in bacteria and plants. The chaperone activity of CSPs is known to disrupt secondary mRNA structure and preserve translation at low temperatures (15). In mammals, YB-1 is extensively studied because of its link with cancer progression and resistance (10,11) and its role in cell development (16). YB-1 putative functions related to nucleic acids encompass mRNA splicing (17), transcription (18), the processing of noncoding RNA (19–22), and DNA repair (23,24). The secretion of YB-1 from mammalian cells has also been reported and may participate in intercellular interactions (25). YB-1 is generally a cytoplasmic protein and a core protein partner of non polysomal mRNA in mammalian cells (26) but it can also be found in the nucleus in cancer cells, a phenotype generally associated with a bad outcome (18). In agreement with the association of YB-1 with non-polysomal mRNAs in the cytoplasm (27), crosslinking immunoprecipitation coupled to sequencing (CLIP) results have indicated a significant binding of YB-1 to coding sequences (CDS) in glioblastoma cancer cell lines. Although a consensus motif has been proposed (UYAUC), the cross links are dispersed across most transcribed coding genes, in agreement with YB-1 being a general mRNP factor (26). More recently, in a large scale CLIP map of multiple RBPs (28), YB-3 was classified as a CDS-binding protein (YB-1 was not analyzed in this study). While YB-1 binds to non polysomal mRNAs, its role in mRNA translation remains debated. Given the inhibition of mRNA translation by recombinant YB-1 in rabbit reticulocyte lysates (29), YB-1 may be considered a translation repressor. On the other hand, the mRNA-unwinding activity of YB-1 has already been advanced to explain the YB-1-dependent translation activation of specific transcripts, such as Snail1 (30) and HIF-α (31) mRNAs. In proliferating cells, YB-1 is generally abundant (32,33) which may contradict the notion that it is a global inhibitor of mRNA translation. Cellular signaling, YB-1 post-translational modification (34,35), and/or protein partners such as Lin28 (36) may either facilitate translation in proliferating cells or maintain YB-1-packaged mRNA in a dormant state, as evidence in testis (37).

Here, we formulated the hypothesis that a putative RNA-unwinding activity of YB-1 prevents mRNAs from forming SGs. However, contradictory results have been reported regarding the role of YB-1 in SG assembly. Most of the data have been obtained in cancer cells upon arsenite treatment, which is the most robust means of driving SGs assembly. In U2OS cells and under certain conditions, YB-1 may positively regulate G3BP-1 expression to promote indirectly SG assembly after arsenite-induced stress (38). In an independent study including U2OS cells, a positive regulation of G3BP-1 expression levels by YB-1 was not confirmed. YB-1 was instead considered dispensable for SG assembly after arsenite treatment but possibly important after ER-stress was induced (39). Interestingly, in the same study (39), a detailed analysis reported a critical role of YB-1 when SGs are formed following the stress-induced cleavage of tRNA into small fragments. Small tRNA fragments can sequester YB-1 to promote the formation of SGs (39) but YB-1 sequestration may in turn also lead to decreased solubility of non polysomal mRNA. In two other studies, arsenite-induced SG assembly was also not significantly affected in cells treated with siRNA to decrease YB-1 levels (40,41). More recently, a recent large-scale analysis seeking to identify positive SG regulators with a CRISPR screen in which YB-1 was included again did not show YB-1 to be among the proteins found important for SG assembly (42). Finally, a study reported increased SG assembly after YB-1 expression was silenced, supporting the idea that YB-1 negatively regulates SG assembly (43).

In addition to experiments based on silencing YB-1 expression, the overexpression of YB-1 inhibited SG assembly in NRK and NG108-15 cells (40,43). The expression of most RBPs such as G3BP-1, TIA-1 (44), TDP-43 (45), and FUS, is either neutral or beneficial to SG assembly in most cases. As overexpressed RNA-helicases such as EIF4A were reported to inhibit SG assembly after arsenite stress (9). An RNA-unwinding activity mediated by YB-1 may thus provide a rational explanation for this specific phenotype.

To explore putative mRNA-unwinding YB-1 activity, we investigated the interaction of YB-1 with short RNA and DNA stem/loops through a combined NMR spectroscopy and molecular dynamics analysis. The results showed clear RNA-unwinding activity due to the loop 3 located in the cold-shock domain, which is long and positively charged. We also identified two YB-1 mutants with decreased ability to unwind RNA in vitro. Then, focusing on the role of YB-1 in SG assembly after arsenite-induced stress, we observed that the overexpression of the RNA unwinding-defective YB-1 mutants less efficiently prevented the formation of SGs than wild type YB-1 in HeLa cells. A possible role of YB-1-RNA-unwinding activity in the inhibition of SG assembly thus appears to be plausible, although alternative interpretations can be proposed notably due to overexpression conditions. However, in agreement with a negative impact of YB-1 in SG assembly, we found that endogenous YB-1 globally increases translation and facilitates SG dissociation during stress recovery in HeLa cells. Interestingly, a strong translation inhibition mediated by the unstructured and self-adhesive C-terminal domain (CTD) of YB-1 was reported *in vitro* (29) but the removal of most of the CTD residues decreased the capacity of YB-1 to prevent SG assembly. In addition, the YB-1 CTD further facilitates the inhibition of SG assembly by YB-1.

We therefore propose a model in which YB-1 prepares non polysomal mRNA for translation through its RNA-unwinding activity. After cellular stress is induced, the large pool of non polysomal mRNA increases the occurrence of RNA:RNA intermolecular interactions in the cytoplasm thereby promoting SG assembly. This occurs when YB-1 is outnumbered by non polysomal mRNA. However, when YB-1 expression is sufficiently high, non polysomal mRNAs no longer form SGs after arsenite stress. Preventing SG assembly may be important to sustain the high proliferative rate of cancer cells and help cells to cope with stress. Accordingly, a recent multitissue transcriptome analysis at the single cell level revealed YB-1 as a key gene in the adaptation of cells to caloric restriction in aging Rattus Norvegicus (46).

## MATERIALS AND METHODS

### Computational methods

#### System preparation and MD simulations setup

The following systems were considered for MD simulations: WT YB-1 protein alone, WT YB-1:RNA/DNA stem loop complexes, where two types of stem loops were considered ACU-4G/ACT-4G and ACU10/ACT10, WT CSD:RNA/DNA stem loop complexes with both stem loop types as above, R97A/K98A YB-1:RNA/DNA stem loop complexes, with ACU-4G/ACT-4G, And K137A/Y138A YB-1:RNA/DNA stem loop complexes, with ACU-4G/ACT-4G.

Protein sequence used for YB-1 is from A45 to Q165 (length 121 a.a.) and for CSD is from A45 to G129 (length 85 a.a.). The length of all RNA/DNA stem loops is of 24 nucleotides.

#### Molecular modeling

The starting 3D coordinates of YB-1 stem loop/RNA models were constructed using as a template the X-ray structure of human Lin28A in complex with let-7f-1 micro RNA pre-element (PDB ID 5UDZ, resolution 2 Å) (47,48). YB-1 structure (A45-Q165) bound to polyC RNA was taken from the trimer complex of YB-1:ssRNA (16-nt C), which is a linear nucleoprotein filament recently published by our group (12) that used the human WT YB-1 NMR structure (position 52–129) as a building block for the cold shock domain (PDB ID: 1H95). Models were constructed by extracting the middle YB-1 structure and superimposing it to Lin28 on the alpha carbons. Lin28 was then removed and the RNA was extended in order to form a total of 24 nucleotides (4 nucleotides forming the loop and 10 bp for the stem). Then the nitrogenous bases were substituted with the stem loop sequences studied here (ACU-4G, ACT-4G, ACU10 and ACT10). In a next step, the CSD:RNA/DNA stem loop complexes were modeled from the YB-1:RNA/DNA structures previously generated by truncating the CTD part (residues 130 to 165). The mutants R97A/K98A and K137A/Y138A were also generated but only for the ACU-4G/ACT-4G stem loops.

#### MD set up

All MD simulations were carried out with GROMACS software package version 2018.2 using the ff03 Amber ‘all atom’ force field with associated nucleic acid parameters and periodic boundary conditions. The protonation states of the residues were adjusted to the PH used in our NMR experiments (6.8). The systems were centered and solvated in a triclinic box of TIP3P water model with 1.4 nm distance between the boundary of the box and the macromolecular complex. A [KCl] of 25mM was used and counter-ions were added to neutralize the system. Each system was first energy minimized using 50000 steps of steepest descent, then heated from 0 to 298 K at constant volume for 500 ps and equilibrated in the NPT ensemble at *p* = 1 atm for 500 ps which was followed by 200 ns of NPT production run. The Velocity Rescaling (with τ = 0.1 ps) and Parrinello-Rahman methods were used for temperature and pressure control, respectively. The equations of motion were propagated with the leap-frog algorithm and the time step was Δ*t* = 2 fs. The particle mesh Ewald (PME) method [was used for electrostatic interactions, with grid spacing of 1.6 Å, a relative tolerance of 10^−5^, an interpolation order of 4 for long-range electrostatics, and a cutoff of 14 Å together with a 12 Å switching threshold for LJ interactions. All covalent bond lengths were constrained with LINCS. Trajectories and geometries have been visualized and represented using VMD. The MD simulations in this work were done using NVIDIA GPU resources owned by Synsight.

### Protein purification

Cells carrying plasmids encoding for wild type and mutant YB1-C (1–180), CSD (50–129), FUS-RRM (275-385) and TDP-43-RRM1-2 (101–277) were grown at 37°C in 2YT-ampicillin medium (1-l culture) (non-labeled proteins) or in minimal medium M9 supplemented with ^15^NH_4_Cl (labeled proteins). When the optical density of the culture reached 0.7, IPTG was added at a final concentration of 1 mM, and growth was continued for 3 h. Cells were harvested and washed with 20 ml of cold 25 mM Tris–HCl buffer, pH 7.4, containing 1 mM TCEP, 1 mM PMSF and EDTA-free protease inhibitor Cocktail (Roche) and 1.5 M KCl (buffer A). The cell pellet was suspended in 10 ml of the same buffer, and cells were disrupted by sonication on ice (Bioblock Vibracell sonicator, model 72412). The resulting suspension was centrifuged at 4°C for 30 min at 150 000 × g in a TL100 Beckman centrifuge. The supernatant was used for purification experiments.

YB-1 proteins were purified under native conditions as previously described (12). The cells were harvested and resuspended in 20 ml of lysis buffer (20 mM Tris–HCl, pH 7.6, 10 mM imidazole, 2M KCl, 0.5 mM DTT, protease inhibitor tables (Roche)). The cell lysate obtained by sonication was cleared by centrifugation at 70 000 × g for 1 h at 4°C. The supernatant was added to Ni-NTA agarose slurry for 30 min at 4°C under agitation and then loaded onto a gravity column. The column was washed with buffer (20 mM Tris–HCl, pH 7.6, 10 mM imidazole, 500 mM KCl, 0.5 mM DTT, 0.5 mM PMSF) and eluted with the same buffer supplemented with 250 mM imidazole. The eluate was then dialyzed against 20 mM Tris–HCl, pH 7.6, 500 mM KCl. Then RNase A treatment was applied to remove all traces of RNA contaminants for 1 h at room temperature under agitation. In a second purification step, the eluate was dialyzed against 50 mM phosphate buffer, pH 6.8, 1 M KCl and concentrated with Spin-X UF concentrators (Corning). Note that we controlled that the structure of His-tagged and non his-tagged protein with or without DNA were similar. The final preparations were stored at –80°C.

TDP-43 fragments were purified basically following the manufacturer's recommendations (Qiagen). Imidazol (10 mM) was added to soluble fractions described above and incubated for 2 h at 4°C with Ni^2+^-NTA-agarose (Qiagen) (20 mg of proteins/ml of resin) pre-equilibrated in buffer A. After incubation, the resin was transferred to an Econo-Pac chromatography column (Bio-Rad). The polymer was then washed extensively with buffer A containing 20 mM imidazole. The elution of the protein was obtained by increasing step by step the concentration of imidazole, from 40 to 250 mM, in buffer A. Pure protein-containing fractions (100–250 mM imidazole) were pooled and incubated with a His_6_-tagged TEV protease to cleave off the His_6_-tag peptide from the target protein. The protease (15 μg) was mixed with 1 mg of target protein (0.5 μM TEV to ∼30 μM protein) in buffer A containing of 1 mM DTT and 1 mM EDTA. All digestions were conducted for 16 h at room temperature. A PD-10 column (GE Healthcare) was used to remove imidazole and to exchange buffer. Then, TEV protease and His_6_-tag peptide from target protein were trapped on Ni-NTA agarose column and target protein was recovered in pass-through (nonbinding) fraction. The protein was concentrated to 2 ml and conserved in 20 mM Tris–HCl buffer, pH 7.4, containing 25 mM KCl and 1 mM TCEP by using a PD-10 column. For NMR experiments, the ^15^N-labeled proteins were stored in phosphate buffer 15 mM pH 6.8 containing 25 mM KCl and 1 mM TCEP by using a PD-10 column (GE Healthcare).

### Nuclear magnetic resonance

Purified ^15^N-labeled protein fragments were incubated with indicated DNA/RNA oligonucleotides (Eurogentec) during 10 min at 25°C. Free and oligonucleotide-bound protein samples were prepared in NMR buffer (25 mM phosphate, pH 6.8, containing 25 mM KCl) supplemented with SUPERase·In RNase inhibitors (ThermoFisher Scientific) for RNA samples. All samples were prepared in a final volume of 60 μl using 1.7 mm diameter capillary tubes (Bruker) and 2,2-dimethyl-2-silapentane-5-sulfonic acid as external reference in pure D_2_O (Eurisotop) for chemical shift referencing.

NMR spectra were acquired on a Bruker AVIII HD 600 MHz spectrometer equipped with a triple-resonance cryoprobe at 298 K. The binding of CSD, YB-1C and TDP-43 to DNA or RNA oligonucleotides was investigated using 2D ^1^H–^15^N SOFAST-HMQC recorded on 50 or 100 μM protein samples at a 1:1 molar ratio. The number of dummy scans and scans was respectively set to 16 and 512. Data were acquired with 2048 points along the direct dimension and with 128 t1 increments with a relaxation delay of 0.2 s. Shaped pulse length and power were calculated by considering an amide ^1^H bandwidth of 4.5 ppm and a chemical shift offset of 8.25 ppm.

NMR assignment: ^1^H and ^15^N chemical shifts of YB-1(1–180) and TDP-43 RRM1-2 residues were assigned using our previous results (12) (YB-1) or previous assignments obtained for the unbound TDP-43 RRM1 and RRM2 (BMRB Entries: 18765 and 19922, 19290).

### Transcription *in vitro*

Reporter mRNA for was transcribed with a SP6-Scribe Standard RNA IVT Kit (CellScript). Polyadenylated *BTF3_Fluc* mRNA was transcribed from pSP36T-5′UTR_BTF3-FLuc-A50 linearized with *Hpa*I. mRNAs were capped using the ScriptCap m^7^G Capping System and the ScriptCap 2′-*O*-methyltransferase enzyme (CellScript) according to the manufacturer's manual.

### *In vitro* translation assays

The translation mixture (10 μl) consisted of 5 μl of nuclease-treated rabbit reticulocyte lysate, 1 μl 10× translation buffer (200 mM HEPES–KOH, pH 7.6, 10 mM DTT, 5 mM spermidine–HCl, 80 mM creatine phosphate, 10 mM ATP, 2 mM GTP and 250 μM of each amino acid), 100 mM KAc, 1 mM Mg(Ac)_2_, 0.15 pmol reporter *Fluc* mRNA, and recombinant YB-1/YB-1C protein. Reaction mixtures were incubated for 25 min at 30°C, and then the luciferase activities were measured using the OneGlo Luciferase Assay kit (Promega). Raw data are shown in Supplementary Table S3.

### Gels mobility shift assays

Indicated amounts of YB-1, YB-1-RK and YB-1-KY (1–180, a.a.) were incubated with 0.16 pmol of 2Luc mRNA (3000 nt) in 20 μl of binding buffer (20 mM HEPES, pH 7.5, 60 mM KCl) at room temperature for 5 min. Complexes were separated in 0.65% agarose gel in 0.5× TAE buffer at 5 V/cm for 20 min and were stained with 0.5 μg/ml ethidium bromide.

### Cell culture and Transfections

HeLa and U2OS cell lines (American Type Culture Collection, USA) were cultured at 37°C in a humidified atmosphere with 5% CO_2_ and maintained in the high glucose formulation of DMEM (Life Technologies) supplemented with penicillin G 100 U/ml, streptomycin 100 μg/ml and fetal bovine serum (FBS) 5% (10% for HeLa cells; Thermo-Fisher).

### Plasmid transfection, siRNA treatment and addback experiments

The cells were grown in 24- or 96-well plates and transiently transfected with plasmids, carrying the studied protein gene, at a final concentration of 1 μg using lipofectamine 2000 (Thermofisher) transfection reagent for 24 h.

Three siRNAs targeting YB-1 expressions were used. Two of them target the coding sequence: siRNA-1: [sense 5′-(CCACGCAAUUACCAGCAAA)dTdT-3′, anti-sense 5′-(UUUGCUGGUAAUUGCGUGG)dTdT-3′]; siRNA-2:[sense 5′-AGUGUAGGAGAUGGAGAAAdTdT-3′, antisense 5′-(UUUCUCCAUCUCCUACACUdTdT-3′]. SiRNA-3 which targets the 3′UTR of YB-1 mRNA was used for the addback experiments [sense 5′-(GAUUGGAGCUGAAGACCUA)dTdT-3′, anti-sense 5′-(UAGGUCUUCAGCUCCAAUC)dTdT-3′]. The negative siRNA (1027310, Qiagen), SiNeg, was applied in the same concentration as YB-1 siRNA. The mix of 1 μg siRNA or siNeg in 300 μl optiMEM with 0.8 μl lipofectamine was left for 20 min at room temperature and added to cells for 3 h, after that the solution was removed and the usual media was added to the well. Control of the efficiency of was performed by immunofluorescence (Figures 8E). We obtained clusters of cells expressing endogenous YB-1 coexisting in the same sample with clusters of cells that displayed a significantly reduced expressing of endogenous YB-1. Only the cells with a low YB-1 expression were retained in the analyzes (Figure 8C).

To add-back the expression of YB-1 (Figure 7, Supplementary Figures S8 and S10), HeLa cells were first treated by siRNA-3 targeting the 3′UTR of endogenous YB-1 for 36 h. Then, HeLa cells were transfected with plasmids to express wild type or mutant YB-1, as indicated. Wild type or mutant exogenous YB-1 expressions obtained by using plasmid transfections are not the targets of siRNA-3.

### *In situ* hybridization

To visualize mRNA using the red channel, after fixation HeLa cells were incubated with oligo-dT-[Cy3], diluted in SSC 2×, 1 mg/ml yeast tRNA, 0.005% BSA, 10% dextran sulfate, 25% formamide, for 2 h at 37°C. Wash steps were performed using 4× and then 2× SSC buffer (0.88% sodium citrate, 1.75% NaCl, pH 7.0). To visualize mRNA in blue color for SGs experiments, the oligo-dT with digoxigenin was used after cells fixation with the same incubation procedure as oligo-dT-Cy3. Then the primary anti-digoxigenin antibodies (mouse, ab420, Abcam) and secondary antibodies (goat anti-mouse, Alexa 350, Invitrogen) were applied to cells according to supplier's protocol.

### SG assay

HeLa cells, transfected with corresponding plasmids for 24 h, were subjected to oxidative stress using arsenite during 1 h at 37°C. The cells were fixed with methanol for 20 min at −20°C, followed with 4% paraformaldehyde for 30 min at 37°C. Immunofluorescence was performed using anti-HA (against overexpressed YB-1, mouse, sc-7392, Santa Cruz Biotechnology), anti-YB-1 (against endogenous protein, rabbit polyclonal, Bethyl Laboratories, Montgomery, USA), anti-FMRP (anti-rabbit, ab 17722, Abcam), anti-G3BP-1 (anti-rabbit, G6046, Sigma)).

Quantifications were performed with Opera Phenix^®^ Plus High Content Screening System (PerkinElmer) in confocal mode. The Harmony v4.8 software was used to detect and measure the number or total area of SGs per cell or the number of SGs per cell (These values are directly accessible by selecting them in the ‘spot analysis’ parameters).

### Screening of the effect of RBP overexpression in SG assembly

To obtain the results presented in Supplementary Figure S6, HeLa cells were transfected with corresponding plasmids for 24 h and were subjected to oxidative stress using 300 μM arsenite during 1 h at 37°C. The cells were fixed with methanol for 20 min at −20°C, followed with 4% PAF for 30 min at 37°C. The staining was performed using anti-HA (YB-1, mouse, sc-7392, Santa Cruz Biotechnology), anti-myc (to detect myc-tagged IGF2BP3) primary antibodies and then secondary antibody (goat anti-mouse/donkey anti-rabbit, Alexa 594, Invitrogen). Other RBPs were visualized with a GFP-tag.

Quantifications were performed by recoding the percentage of cells harboring SGs from fluorescence images.

### Cellular translation assays

The cells were treated with puromycin (10 μg/ml) for 10 min prior to fixation after washing out puromycin. Cell were fixed with 4% PAF for 30 min at 37°C and subjected to immunoblotting using puromycin antibody (Merck, MABE343) or beta-actin antibody (Sigma, A2228) as described previously. For the negative control, cells were treated with cycloheximide (100 μg/ml) prior to the addition of puromycin. The anti-puromycin fluorescence in the cytoplasm was detected automatically using the Opera Phenix^®^ Plus High Content Screening System (PerkinElmer). The cell cytoplams were detected automatically using the Harmony v4.8 software.

### DNA-melting activity assay

Before assay DNA oligonucleotide 5′FAM-GGGGGGGGTTAACCCCCCCC-BHQ1-3′ (DNA1) was incubated 5 min at 98°C and slowly chilled to 20°C. The reaction mixture of a 20 μl final volume contained 130 mM KCl, 10 mM HEPES–KOH (pH 7.6), 20 pmol of the DNA1 oligonucleotide and 200 pmol of protein (YB-1, or YB-1 fragment (1–180), or YB-1 fragment (1–180) mutant forms). The control experiment was performed without proteins. All reaction mixtures were incubated at 30°C in a DTlite real-time PCR system (DNA-Technology, Russia) to detect fluorescence every 5 s for 5–25 min. To plot the graph, the first fluorescence value detected by the real-time PCR system was subtracted from the subsequent ones as background.

## RESULTS

### YB-1 unwinds DNA/RNA stem/loops *in vitro*

**Figure 1.**
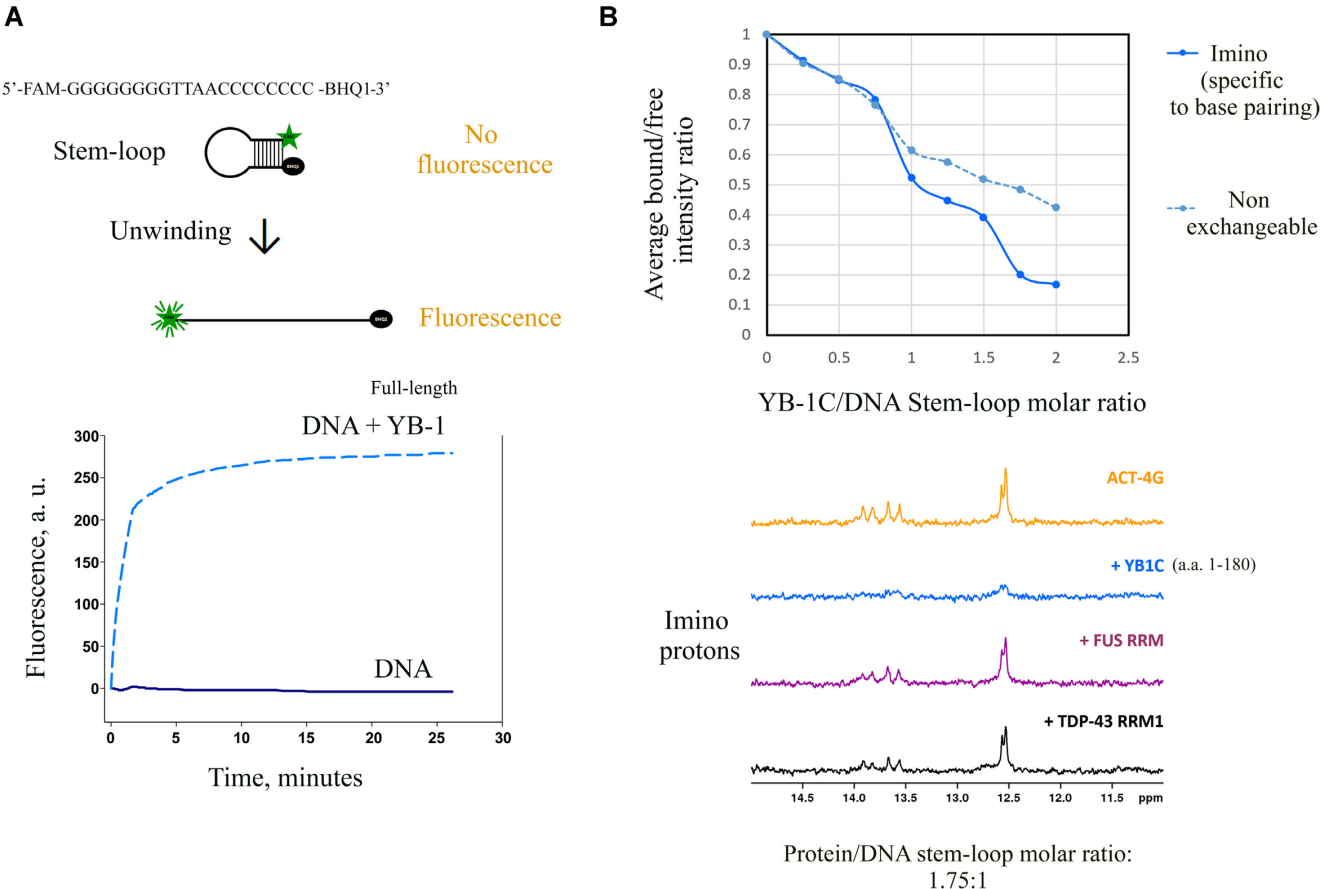
YB-1 destabilizes DNA stem/loops *in vitro*. (**A**) Full-length YB-1 melts short DNA duplex. DNA oligonucleotide 5′FAM-GGGGGGGGTTAACCCCCCCC-BHQ1-3′ (*T*_m_ ∼ 70°C) was incubated 5 min at 98°C and slowly chilled to 20°C. After YB-1 protein (dashed line) or buffer (solid line) addition, reaction mixtures were incubated at 30°C in a real-time PCR system to detect fluorescence every 5 s for 26 min. (**B**) Upper panel: NMR analysis of the average bound:free intensity ratio of the ACT-4G stem/loop (ACAGACAGAACCCCTTCTGTCTGT, *T*_m_ ∼ 60°C) imino and non-exchangeable protons as a function of protein:DNA concentration ratio. 1D ^1^H NMR spectra at 25°C of 50 μM ACT-4G DNA stem free (in orange) and in presence of YB-1C (in blue). The decrease of peak intensity with increasing concentration of YB-1 is more marked for imino than non-exchangeable protons. Lower panel: Imino region of the 1D ^1^H NMR spectra in the presence of YB-1C (in blue), FUS RRM (in purple) or TDP43 RRM1 (in black) at a protein:DNA concentration ratio of 1.75:1. The decrease of imino signals with YB-1C corresponds to an increase of imino proton exchange in agreement with a stem/loop opening.

YB-1 possesses three distinct domains (10,14): (i) an unstructured alanine/proline-rich domain which may interact with protein partners, (ii) a highly conserved β-barrel structure, i.e., the cold-shock domain (CSD) that binds to RNA and DNA and (iii) a long unstructured C-terminal domain (CTD) that harbors well separated clusters of negatively and positively charged residues that may be involved in self-adhesive interactions (49). The melting of RNA secondary structures (50) and the separation of DNA strands by YB-1 have been observed *in vitro* (51). To confirm that YB-1 unwinds the secondary structures of nucleic acids *in vitro*, a DNA stem/loop with a quencher and a fluorophore at each extremity and a short loop that could accept only one protein was used to probe a putative unwinding activity (Figure 1A). The results of the fluorescence assay clearly showed a significant increase in fluorescence intensity in the presence of YB-1 revealing an opening of the DNA stem/loop by full-length YB1.

To examine the molecular interactions critical for the unwinding of RNA secondary structure, we performed an extensive NMR analysis with a truncated form of YB-1 (1–180, a.a., also called YB-1C). With its N-terminus (1–50, a.a.), CSD (51–128, a.a.), and short CTD (129–180, a.a.) including a cluster of arginine residues, YB-1C was to date the longest YB-1 fragment amenable to NMR spectroscopy. We then analyzed the unwinding activity of YB-1C by NMR spectroscopy at different YB-1C concentrations. The peak intensities of imino protons that are specific to DNA/RNA base pairing were probed using a short stem/loop (ACAGACAGAA**CCCC**TTCTGTCTGT, called ACT-4G, *T*_m_ ∼ 60°C). A marked decrease in peak intensities was observed at high YB-1C concentrations (Figure 1B). In addition, the imino protons displayed a steeper slope than non-exchangeable protons which are not specific to base pairing. These results therefore indicated a destabilization of DNA stem/loop in addition to an increase in molecular weight (Supplementary Figure S1a, b). The peak intensities of the imino protons were also measured in the presence of two different RNA-recognition Motifs (RRMs), the RRM of FUS and RRM-1 of TDP-43. Despite the presence of CSPs indicating the binding of the RRMs to the DNA stem/loop (data not shown), the peak intensities were not significantly affected (Figure 1B).

According to this analysis, the DNA stem/loop destabilization was more pronounced at high YB-1 concentrations (Supplementary Figure S1b). The binding of several YB-1 proteins was probably required to secure the opening of the 4 C-G stem:loop. However, even at a YB-1C:DNA molar ratio lower than 1:1, YB-1C has the capacity to form a multimer because of its cooperative binding to ssDNA (12,52).

**Figure 2.**
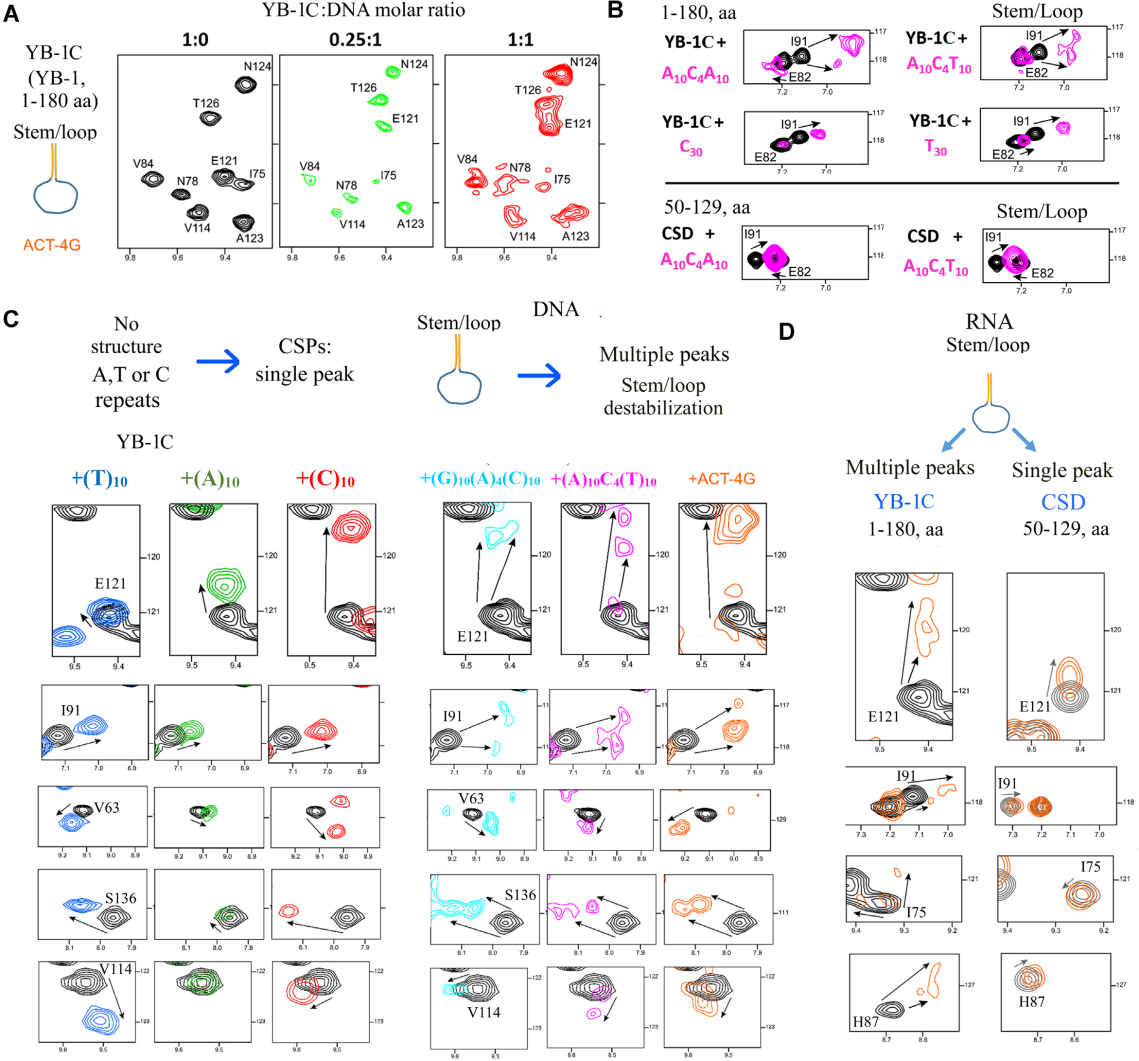
YB-1C destabilizes RNA/DNA stem loop but not CSD alone. (**A**) Two-dimensional ^1^H–^15^N HSQC spectra of YB-1C (1–180 a.a.) in free state or in presence of the ACT-4G stem/loop (ACAGACAGAACCCCTTCTGTCTGT, *T*_m_ ∼ 60°C) at different YB-1C:DNA molar ratios. Note the appearance of multiple peaks at a 1:1 molar ratio. (**B**) As observed with I91, a representative residue, multiple peaks are observed when YB-1C interacts with a stem/loop or an unstructured hetero-nucleotide ssDNA mimicking an open stem/loop but not with a long homo-nucleotide ssDNA. Only single peaks were also detected with CSD alone (50–129 a.a.) that cannot multimerize like YB-1C. (**C**) NMR spectra of YB-1C (1–180 aa) in free state or in presence of indicated unstructured or structured DNA oligonucleotides. Representative residues experiencing CSPs with multiple peaks in the presence of DNA stem/loops are shown. The experiments were performed at 25°C, a temperature well below the Tms of the DNA stem/loop (ACT10, *T*_m_ ∼ 49°C; ACT-4G, *T*_m_ ∼ 60°C). (**D**) Two-dimensional ^1^H–^15^N HSQC spectra of YB-1C (1–180 a.a.) and the CSD alone (50–129 a.a.) in free state or in presence of the ACU-4G RNA stem/loop (*T*_m_ ∼ 60°C).

We then considered the chemical shift perturbations (CSPs) of ^15^N-labeled YB-1 residues induced by DNA/RNA stem/loops. In the presence of the ACT-4G stem/loop, we noticed the presence of multiple peaks and peak broadenings (Figure 2A, B)). Multiple peaks are typical of a slow exchange regime between different states suggesting an interaction of at least two YB-1 proteins per stem/loop. The destabilization of stem/loops generates additional ssDNA binding sites presumably available for CSD. Accordingly, multiple peaks were detected in the conserved CSD residues interacting with nucleic acids (V63, I91 and E121 (Supplementary Figure S2b)). Similar results were obtained in the presence of an RNA stem/loop (ACAGACAGAA**CCCC**UUCUGUCUGU, *T*_m_ ∼ 60°C) (Figure 2D). To test whether more than one YB-1C can interact with the same stem/loop, we analyzed the interaction of YB-1C at a low YB-1C:DNA molar ratio (at 0.25:1) when the occurrence of multiple bindings to the ACT-4G stem/loop should decrease. Multiple peaks indeed disappeared at a low YB-1C:DNA molar ratio (0.25:1, Figure 2A). We also analyzed the interactions of YB-1 with two other DNA stem/loops that had a short loop, G_10_–A_4_–C_10_ (*T*_m_ ∼ 85°C), A_10_–C_4_–T_10_ (*T*_m_ ∼ 49°C). Multiple peaks were again detected at a 1:1 YB-1C:DNA molar ratio.

To further test whether the appearance of multiple peaks is due to stem/loop destabilization, we performed a series of experiments. First, we controlled the presence of single peaks when YB-1C interacted with unstructured A, T or C homo-oligonucleotides (A_10_, T_10_, C_10_; Figure 2C, D). G_10_ was not considered because of the formation of G-quartets. In addition, single peaks were detected when multiple YB-1C proteins oligomerized in the presence of 30 nt-long homo-nucleotides such as poly(C) or poly(T), as shown previously (12), probably because each YB-1C protein interacted with the same nucleotides in the multimer (Figure 2C). However, an unstructured A_10_–C_4_–A_10_ ssDNA mimicking an open A_10_–C_4_–T_10_ stem/loop induced the appearance of multiple peaks (Figure 2B, C, Supplementary Figure S2a). The binding of multiple YB-1 proteins to A_10_–C_4_–A_10_ may explain this behavior since YB-1 proteins in the multimer should not interact with the same nucleotides, in contrast with YB-1 interactions with long homo-oligonucleotides (Figure 2B, C).

We also performed a similar experiment with an isolated CSD (50–129, a.a.) that was unable to form multimers with ssDNA/RNA under our experimental conditions (12). Multiple peaks in NMR spectra failed to appear in CSD residues (Figure 2B, D). One CSD per RNA stem/loop most probably binds to the RNA loop without opening the stem. Single peaks were also detected in the presence of the A10–C4–T10 stem/loop with the tandem RRM1-2 of TDP-43 (Supplementary Figure S2c).

Altogether, the disappearance of imino protons from DNA stem/loops, the CSPs of YB-1C residues and, complementary fluorescence assays showing the DNA-melting activity of YB-1 support a destabilization of nucleic acid stem/loops by YB-1.

### CSD Loop 3 and CTD residues interact and thus recruit many positively charged residues near stem in a YB-1:stem/loop complexes

While seeking to determine how YB-1 may unwind RNA, we noticed the presence of 4 positively charged residues in the CSD loop 3 (**KK**NNP**RK**YL; 92–100, a.a.) in Y-Box protein family members, but not in other cold-shock proteins in humans (such as Lin28 or CSDE1) which retained our attention. In addition, we noticed that loop 3 in Y-box proteins is the longest among the cold-shock proteins (Supplementary Figure S4). To understand the role of a long CSD loop 3, we examined the crystal structure of Lin28, complexed with let-7 (47) a micro-RNA with a stem/loop structure, and noticed the location of the CSD loop 3 with respect to the stem. Assuming a similar binding of YB-1 and Lin28 CSDs on basis of their high structural homology, the additional positively charged residues found in the CSD loop 3 of YB-1 may interfere with the base-pair stability between complementary strands in the stem, possibly leading to the possible destabilization of the RNA stem/loop.

**Figure 3.**
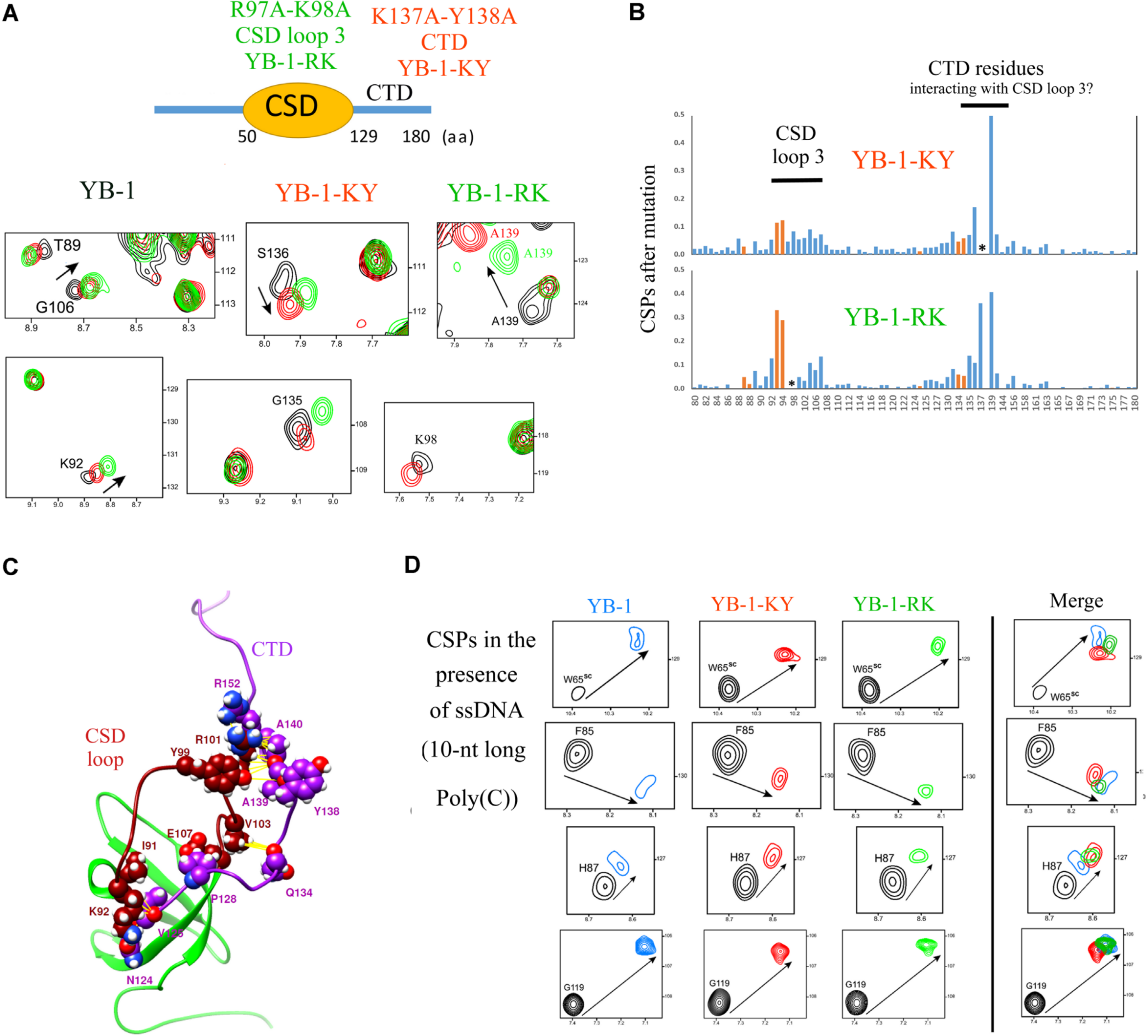
The CSD loop 3 interacts with residues located in the CTD to orient the CTD. (**A**) CSPs of some YB-1 residues located in the CTD or near the CSD loop 3 for the two mutants, YB-1-RK (R97A/ K98A), YB-1-KY (K137A/Y138) in the absence of nucleic acids. (**B**) Histograms displaying the CSPs for the two mutants compared to wild type YB-1 (Supplementary Figure S3c, NMR spectra). A putative interaction between CTD and CSD loop 3 is clearly detected. Red bar, side-chain. *, mutated residues. (**C**) MD model of the interaction between residues located in the CSD loop 3 and the CTD. (**D**) CSPs of conserved residues interacting with nucleic acids in YB-1, YB-1-RK and -KY in the presence of 10-nt long poly(C) oligonucleotides. The mutations do not significantly affect the binding of the CSD to ssDNA.

To determine whether CSD loop 3 plays a key role in RNA-unwinding, we generated a mutant, YB-1-RK, by substituting two positively charged residues (R97 and K98) with alanine residues. Given the central location of R97 and K98 in loop 3, the chances of altering the stable β-barrel structure through their mutation into alanine are limited. To analyze the impact of these mutations in the YB-1 structure, ^15^N-labeled wild type YB-1 (1–180, a.a.) and the R97A/K98A mutant were investigated by NMR spectroscopy in liquid at 25°C (Supplementary Figure S3b). Unexpectedly, the double mutation, R97A/K98A, had a marked impact on the chemical shifts (CSPs) of the residues located in a segment of the CTD (130–140, a.a.), although the spatial proximity of these residues with CSD loop 3 is not intuitive at first sight (Figure 3A, B and Supplementary Figure S3c). We then generated a molecular dynamics (MD) model of YB-1C alone (Figure 3C, 1–180 a.a.), as described in the Methods. Several interaction pairs displayed a low free energy (G106-Q134, E107-Q134, R101-R152, Y99-Y138, Y99-A139, Table 1) suggesting a putative interaction between CSD loop 3 and the CTD segment (135–140, a.a.). Consistent with this finding, an interaction between CSD loop3 and the CTD in a shorter YB-1 mutant (51–140 a.a.) has been recently proposed (53), although this mutant is probably too short to fully capture the dynamics of the CTD segment. To disrupt the putative interaction between CSD loop 3 and CTD, we prepared a new mutant (YB-1-KY) with two mutations, K137A/Y138A, in a central location in the CTD segment that interacts with CSD loop 3. Strikingly, the CSPs observed for the YB-1-KY and YB-1- RK mutants were very similar to the CSPs of wild type YB-1, which indicates that the double mutation, K137A/Y138A, most probably disrupts the interaction between the CTD segment with CSD loop 3 (Figure 3A, B). The CTD residues involved in the interaction with CSD loop 3 are also highly conserved across species (Supplementary Figure S4), in contrast to the remainder of CTD sequences. Only the presence of positively charged residues is conserved. This residue conservation is an additional argument for an interplay between CSD loop 3 and the CTD, which could also initiate an interaction between the additional arginine residues in the CTD (R142, R146-R147, R150-153) with the stem (Figure 3A, B), thus further contributing to the destabilization of base pair interactions (Supplementary Table S1).

**Table 1.** Interaction energies between the most relevant YB-1 pair residues from CSD loop 3 and CTD. Energies were averaged over the whole MD trajectory (100 ns)

Residue pair		
CSD loop	C-ter	Δ*E* (kJ/mol)
I91	N124	–1.8
	V125	–18.7
K92	N124	–18.8
	V125	–14.2
Y99	Y138	–16.2
	A139	–12.4
R101	A140	–6.3
	R152	–20.0
V103	Q134	–11.0
G106	Q134	–20.0
	G135	–3.6
E107	P128	–20.5
	Q134	–52.5
Total		–216.0

### Identification of two mutants, YB-1-RK and -KY with a reduced efficiency in destabilizing DNA/RNA stem/loops

To investigate the molecular mechanisms by which CSD loop 3 and the CTD promote the unwinding of nucleic acids, we performed a combined NMR spectroscopy and MD analysis with different DNA/RNA stem/loops in interaction with wild type YB-1C or YB-1C mutants, YB-1-RK and YB-1-KY. Prior to this analysis, the capacity of YB-1C mutants to bind unstructured ssDNA was controlled (Figure 3D). We noticed that all the conserved residues in the β-barrel display strong chemical shifts that are very similar to those observed with wild type YB-1 (H87, F85, W65 and G119) in the presence of unstructured ssDNA. CSPs for residues located near loop 3 or in the CTD residues also showed similar shifts, except for the mutated residues or those near the mutated residues. In summary, the results indicate unaffected binding of YB-1-RK and -KY CSD residues to unstructured ssDNA compared to wild type YB-1 (Figure 3D).

**Figure 4.**
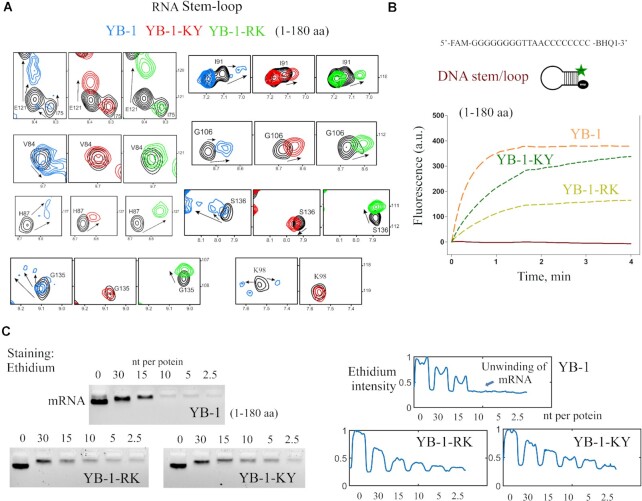
YB-1-RK and -KY do not destabilize RNA stem/loops, in contrast with wild-type YB-1. (**A**) CSPs of conserved residues interacting with RNA, located in the CTD or nearby the CSD loop after the interaction of wild type and mutant YB-1 with RNA stem/loop (*T*_m_ ∼ 60°C). *T* = 25°C. (**B**) YB-1C mutants (1–180 a.a.) are less effective in DNA duplex melting than YB-1C. DNA oligonucleotide 5′FAM-GGGGGGGGTTAACCCCCCCC-BHQ1-3′ (DNA1) was incubated for 5 min at 98°C and slowly chilled to 20°C. After protein (dashed line) or buffer (solid line) addition, reaction mixtures were incubated at 30°C in a real-time PCR system to detect fluorescence every 5 s for 4 min. Orange dashed line: YB-1C, green dashed line: YB-1-KY, light green dashed line: YB-1-RK. (**C**) Left panel: Electrophoretic mobility of mRNA in the presence of wild type and mutant YB-1 (1–180, a.a.). Wild type YB-1 progressively decreases ethidium fluorescence of mRNA bands due to mRNA unwinding but to lesser extent with YB-1 mutants. Right panel: Intensity of ethidium fluorescence for the indicated bands. Another independent experiment is shown in Supplementary Figure S3d.

We then analyzed the interactions of YB-1-RK and -KY with an RNA stem/loop, ACU-4G, by NMR spectroscopy. Significant differences in the CSPs were detected as multiple peaks were no longer present for the conserved RNA-interacting residues in YB-1-RK and -KY in contrast with wild type YB-1 (Figure 4A, V84, H87, I91, E121, G106, G135, S136). The absence of multiple peaks indicates a single environment for the CSD residues of YB-1-RK and -KY, most probably because of their interactions with the RNA loop that do not destabilize the stem. To verify this supposition, we analyzed the opening of DNA stem/loop with a florescence assay. While YB-1C significantly increased the fluorescence intensity due to the opening of the stem/loop (as observed with full length YB-1, Figure 1A), both YB-1-RK and -KY increased the fluorescence intensity to a lesser extent than YB-1C, in agreement with an impaired unwinding of the DNA stem/loop by the YB-1 mutants (Figure 4B). We also analyzed the fluorescence of ethidium bromide (EtBr) in mRNA:protein complexes formed in the presence of a long mRNA (Figure 4C, 2Luc mRNA, 3000 nt). The intercalation of EtBr in double stranded nucleic acids results in a high-fluorescence yield. Accordingly, the unwinding of mRNA secondary structures would reduce EtBr fluorescence. We previously showed that YB-1C (1–180, a.a.), but not HuR, an RNA-binding protein with RRM domains, significantly reduced the intensity of EtBr fluorescence in similar way in *Escherichia coli* SSB, which forms ssDNA nucleoprotein filaments (12). We first noticed that wild type and mutant YB-1 induced a similar decrease in electrophoretic mobility, suggesting a similar affinity for mRNA. Low YB-1C concentrations, (>10 nt per protein), mutated or not, considerably reduced EtBr fluorescence, most probably due to the dissociation of the very short RNA stems that cannot resist the binding of the CSD to ssRNA. However, at higher YB-1C concentrations (<10 nt per protein), wild type YB-1C reduced EtBr fluorescence to a greater extent than YB-1-RK or-KY (YB-1C with mutations). This pattern most probably reflects the unwinding of strong stem/loop by YB-1C and to lesser degree by YB-1-RK and -KY (Figure 4C).

### The molecular mechanism behind the destabilization of RNA stem/loop by YB-1

**Figure 5.**
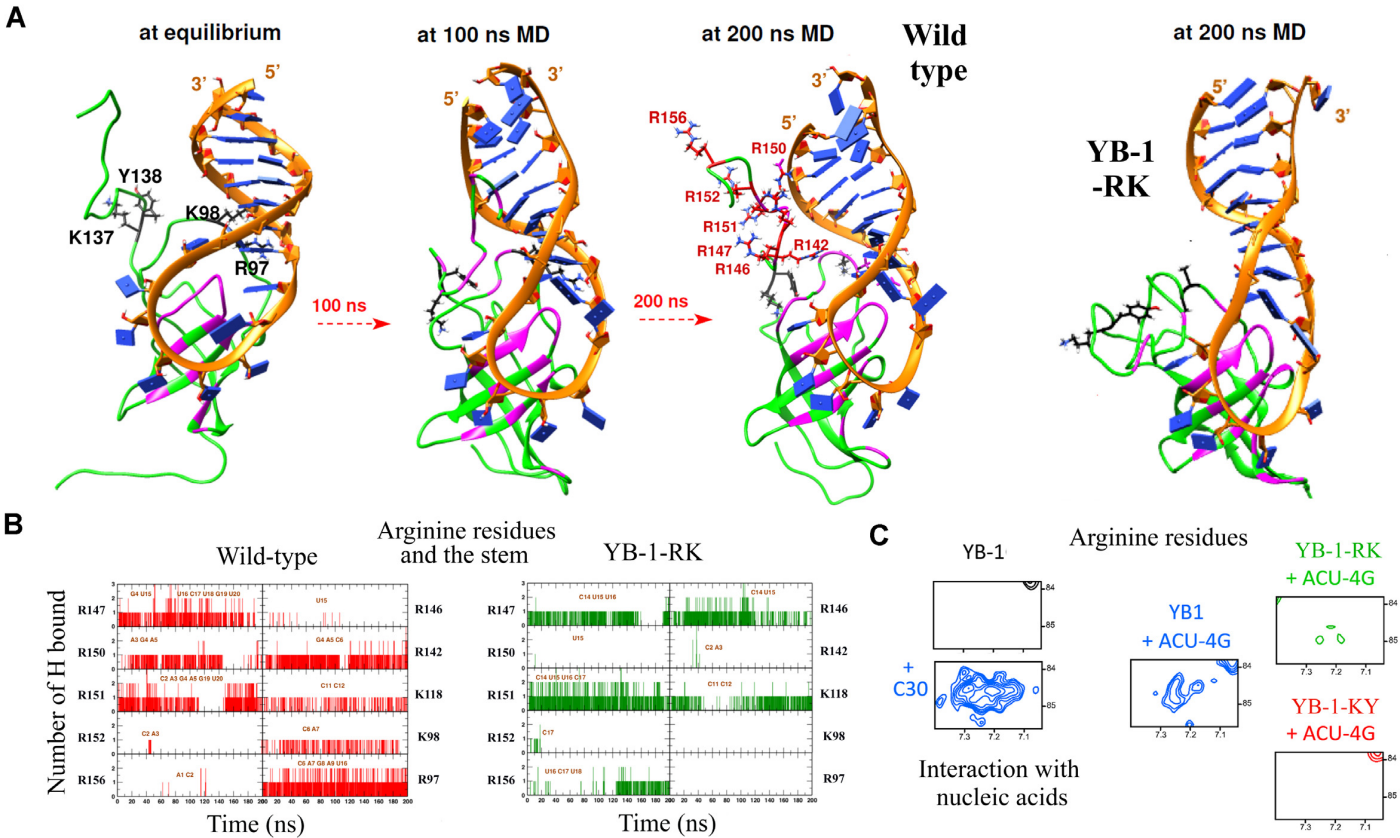
Molecular mechanism of RNA/stem loop destabilization by YB-1. (**A**) MD time series of wild type YB-1 interacting with an RNA-stem/loop. R97/K98 interacts with the beginning of the stem. CTD arginine residues are then brought in the vicinity of the stem. In YB-1 RK, arginine residues are located away from the stem. (**B**) Representation of H-bonds between CTD arginine residues and the stem versus time according to MD simulations. (**C**) Hϵ/Nϵ resonance peaks of arginine residues mostly located in the CTD appeared only after their interaction with single stranded nucleic acids and in the presence of an RNA stem/loop for wild type YB-1 but poorly for YB-1-RK and -KY.

We then considered the molecular mechanism leading to the RNA-unwinding activity mediated by YB-1. To this end, a MD model of YB-1 complexed to the ACU-4G RNA stem/loop was generated (Figure 5A and Supplementary Figure S5a). Using the structure of Lin28 associated to let-7 microRNA as a starting position, the energies averaged over the whole MD trajectory (200 ns) indicate that wild type YB-1 significantly destabilized the base pairing interactions within the stem (Supplementary Table S1). Based on these MD data, the RNA stem destabilization is caused by the CSD loop 3 localized in the vicinity of the stem with a noticeable contribution of R97 (Supplementary Figure S5c and Supplementary Table S2). An additional contribution comes from arginine residues, R142, R147, R150 and R151 located in the CTD that interact with the stem. In contrast, in YB-1-RK, the interactions of the loop 3 and CTD arginine residues with the RNA stem decrease dramatically, preserving the stability of the stem (Figure 5B). The unwinding activity of YB-1-KY is also reduced but to a lesser extent in the presence of YB-1-RK since CSD loop3 remains in the vicinity of the stem in YB-1-KY. However, certain arginine residues in YB-1-KY such as R142 and R150 interact only weakly with the stem, possibly reducing the capacity of the CTD to unwind RNA (Figure 5B, Supplementary Figure S5b, d and Table 2 and Supplementary Table S1). To confirm the role of the CTD arginine residues, we analyzed the interactions of arginine residues with nucleic acids evident in the NMR spectra. While arginine side chains in the unstructured CTD (Hϵ/Nϵ peaks) were not detected due to rapid exchange with water, their interactions with RNA/DNA phosphates reduced the solvent-exchange dynamics, enabling their detection in NMR spectra (Figure 5C). When YB-1 oligomerizes along the 30 nt-long ssDNA, the arginine residues of CTD interact with nucleic acid backbones to provide an electrostatic bridge between consecutive CSDs. However, in the presence of an RNA stem/loop, the Hϵ/Nϵ peaks of the arginine residues were scarce in the YB-1-RK and -KY mutants and less intense than those of wild type YB-1 (Figure 5C). The electrostatic interaction of CTD arginine residues with nucleic acids may therefore be less effective in the two YB-1 mutants.

**Table 2.** Hydrogen bonds occupancy (%) for arginine and lysine residues of CSD loop 3 and CTD in interaction with the RNA stem during 200 ns of MD simulation, for the WT, R97A/K98A and K137A/Y138A complexes. Hydrogen (H) bonds were counted between donors (D) and acceptors (A) provided that the D-A distance is less than 3.0 Α and the D-H-A angle is less than 20 degrees

H-bond occupancy (%)				
RNA base pairs	Residues	WT	R97A-K98A	K137A-Y138A
**A1**	**R152**			1.2
	**R156**	0.1		
**U24**	**R156**			0.65
	**Total**	0.1	0	1.85
**C2**	**R142**		0.15	0.15
	**R150**			1.64
	**R151**	15.84		
	**R152**	9.15		6.08
	**R156**	0.5		
**G23**	**R156**			3.59
	**Total**	25.49	0.15	11.46
**A3**	**K98**			0.6
	**R142**		0.2	
	**R150**	1.75		2.8
	**R151**	9.14		
	**R152**	0.5		13.19
**U22**	**R156**			2
	**Total**	11.39	0.2	18.59
**G4**	**K98**			2.7
	**R142**	1.2		
	**R147**	0.2		
	**R150**	16.53		
	**R151**	11.94		
	**R152**			11.14
**C21**	**R156**			2.4
	**Total**	29.87	0	16.24
**A5**	**R97**			3.1
	**K98**			0.8
	**R142**	38.16		
	**R150**	0.15		
	**R151**	0.2		
	**R152**			0.05
**U20**	**R147**	0.45		
	**R151**	2.85		
	**R152**			0.55
	**R156**			4.25
	**Total**	41.81	0	8.75
**C6**	**R97**	0.2	4.69	
	**K98**	1.9		
	**R142**	0.1		
**G19**	**R147**	1.75		
	**R150**		0.05	
	**R151**	1.9	1.5	
	**Total**	5.85	6.24	
**A7**	**R97**	3.9	12.99	
	**K98**	9.99		
**U18**	**R147**	1		
	**R151**			16.23
	**R156**		1.7	
	**Total**	14.89	1.7	29.22
**G8**	**R97**	24.83		11.54
**C17**	**R97**			1.15
	**R147**	10.84		
	**R151**		16.98	1.65
	**R152**		1.1	
	**R156**		2.4	
	**Total**	35.67	20.48	14.34
**A9**	**R97**	27.12		0.25
**U16**	**R97**	0.05		
	**R147**	7.69	0.3	0.05
	**R151**		0.1	
	**R156**		8.89	
	**Total**	34.86	9.29	0.3

Taken together, the structural data confirmed a destabilization of DNA/RNA stem/loops by the positively charged residues located in CSD loop 3 and the CTD, when the CTD is ideally oriented through its interaction with CSD loop 3. The data also confirmed the RNA-unwinding deficiency of YB-1-RK and YB-1-KY.

### Defective RNA-unwinding activity impairs the negative regulation of SG assembly by YB-1 and the positive regulation of mRNA translation

We next considered whether YB-1-mediated mRNA-unwinding activity may negatively regulate SG assembly in cells. We first screened the formation of SGs in HeLa cells overexpressing different RNA-binding proteins after arsenite treatment, which is the most robust means to trigger the formation of SGs in most cells and the best documented SG assembly mechanism. *In situ* hybridization with a poly(dT) probe was used to detect SG in cells. In HeLa cells overexpressing GFP-tagged YB-1, IGF2BP3, TDP-43, G3BP-1, KHSRP, LARP6, WBP11, PUM2, CIRPB, Lin28 or CSDE1, we found that only YB-1 or IGF2BP3 overexpression affected SG assembly (Supplementary Figure S6a,b and S6a). Interestingly, an interaction between YB-1 and IGF2BP3 in primary human and mouse acute myeloid leukemia cells was recently reported (54). However, given the abundance of YB-1 in the cytoplasm (80-fold greater abundance than IGF2BP3 in HeLa cells (55)) and its preferential binding to mRNA, YB-1 is a better candidate than IGF2BP3 for preventing the global recruitment of non polysomal mRNA into SGs. The inhibition of SG assembly in HeLa cells overexpressing YB-1 was also observed by using G3BP-1 and FMRP (56), two cytoplasmic RBPs, as SG markers (Supplementary Figure S7a).

**Figure 6.**
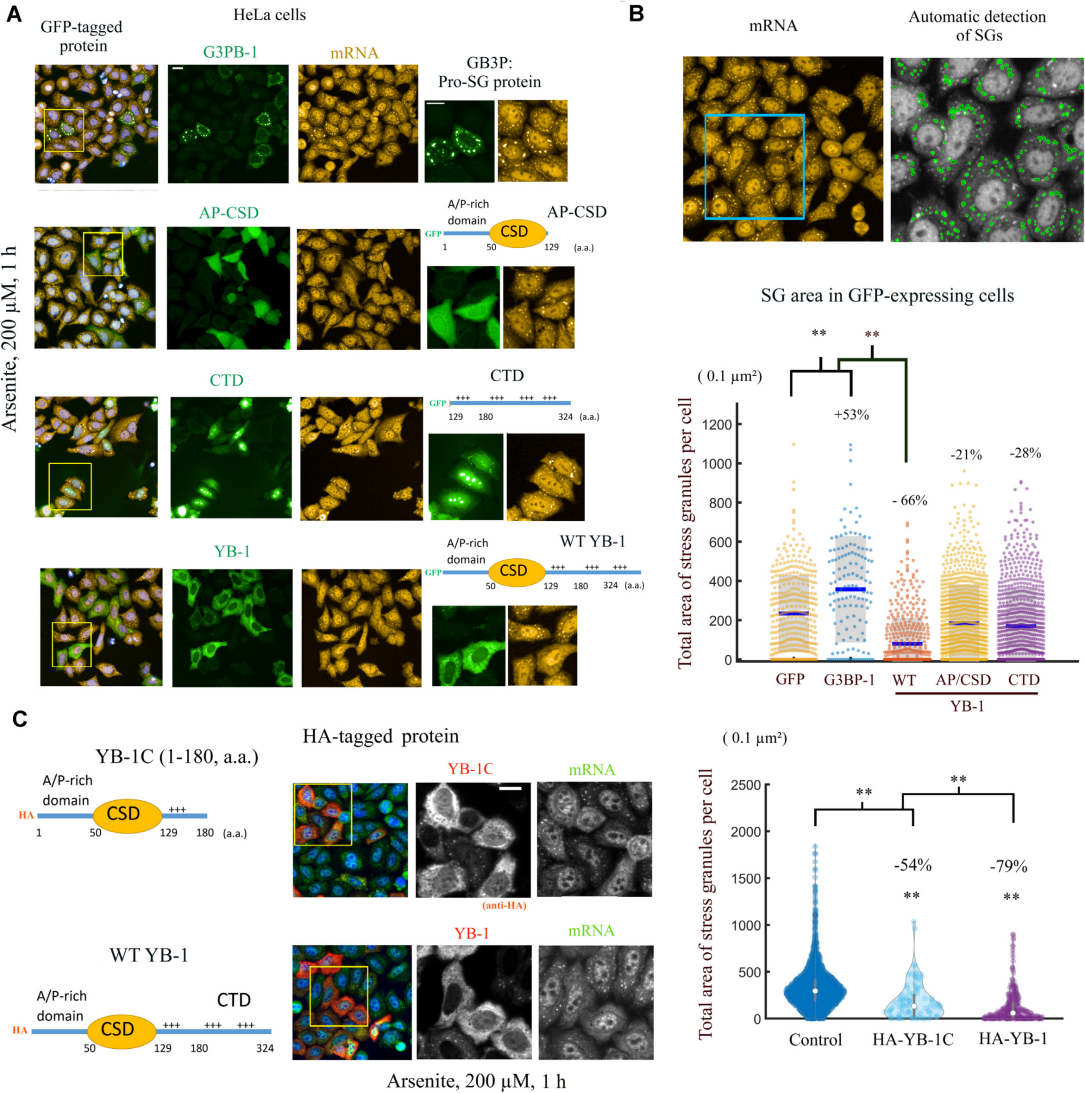
The overexpression of YB-1 in cells prevents SG formation. (**A**) G3BP-1, full length YB-1, AP-CSD (1–129 a.a.) and CTD (129–324 a.a.) were expressed in HeLa cells with a GFP tag (N-terminus) prior to arsenite treatment (200 μM, 1 h). SGs were detected with a fluorescent poly(T) probe that targets poly(A) mRNA. Representative images are shown. We noticed a partially nuclear localization of CTD. Scale bar: 40 μm. (**B**) Analysis of SG assembly in HeLa cells overexpressing GFP-tagged proteins as indicated. Upper panel: a representative image showing the automatic detection of SGs by using a HCS imager. Lower panel: Single cell analysis of the total area of SG per cell for indicated conditions, a quantitative indicator of SG assembly. Cells expressing GFP were selected by using the same threshold value of the mean GFP fluorescence in the cytoplasm. %: difference in mean total SG area per cell compared to cells expressing GFP alone. **P < 0.01; *t*-test with two tails. Each dot represents one cell. (**C**) HeLa cells expressing indicated HA-tagged (N-terminus) proteins (YB-1C or YB-1) were treated with arsenite to generate SGs. The total area of SGs per cell was measured as explained in (B). **P < 0.01; *t*-test with two tails. %: difference in median total SG area per cell versus control. Scale bar: 40 μm.

We then tested different truncation mutants to determine which YB-1 domain is critical for the inhibition of SG assembly (Figure 6A). To this end, the total area of SGs per cell, which is an unbiased and measurable parameter to estimate the level of SG assembly per cell (Figure 6A), was quantified in HeLa cells expressing GFP-tagged proteins including full length or YB-1 truncated proteins. Compared to the effect of GFP alone, G3BP-1 promoted the formation of SGs. In contrast, full length YB-1 led to a striking decrease in the total area of SGs per cell. However, no significant impairment in SG assembly was measured when the YB-1 CTD was totally removed (A/P-CSD, 1–129 a.a.) (Figure 6B). The unstructured CTD (129–324, a.a.) was also not sufficient to inhibit SG assembly (Figure 6B). As both CSD and CTD are required for the inhibition of SG assembly, we tested whether YB-1C (1–180 a.a.) can prevent SG assembly, even with its short CTD (Figure 6C). The results indicated a significant decrease in total SG area in HeLa cells expressing YB-1C. As a short CTD is also required to unwind RNA *in vitro* (Figure 2D), the negative regulation of SG assembly by YB-1 correlates with the capacity of YB-1 to unwind RNA. In addition, while the YB-1 CTD is self-adhesive and critical for YB-1 oligomerization *in vitro*, full length YB-1 further decreased the total SG area per cell compared to YB-1C (Figure 6C). Because SG composition and property varies by stress type (57), we sought to determine whether the overexpression of YB-1 in HeLa cells prevents SG assembly after a combined puromycin/hydrogen peroxide treatment. Puromycin causes premature chain termination, which facilitates the appearance of SGs in most hydrogen peroxide-treated HeLa cells (Supplementary Figure S7b). Again, YB-1 overexpression in HeLa cells strongly inhibits SG assembly. In addition, we tested the inhibition of SG assembly by expressing YB-1 in another cell line, U2OS cells (Supplementary Figure S7c). We observed a marked decrease in SG assembly in U2OS cells overexpressing YB-1.

**Figure 7.**
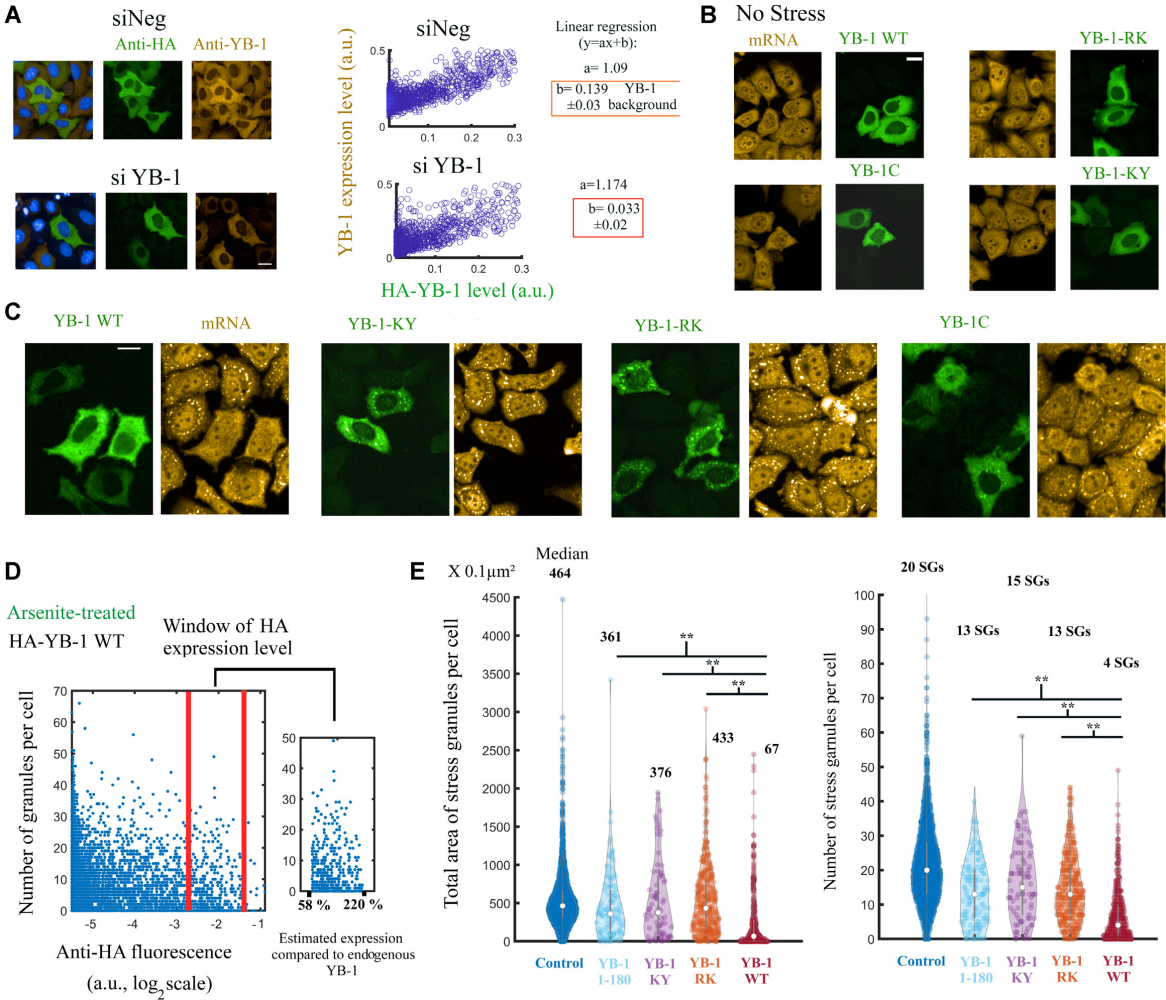
RNA unwinding-defective YB-1 mutants are less potent than wild type YB-1 in preventing SGs assembly. (**A**) Left panel: representative images of HeLa cells expressing HA-tagged YB-1 that were pretreated with control siRNA (siNeg) or siRNA targeting the 5′UTR of YB-1 mRNA. Anti-YB-1 and anti-HA antibodies were used to quantify the expression level of total YB-1 and HA-tagged YB-1. Right panel: Analysis at the single cell level of the expressions of total YB-1 and HA-Tagged YB-1. The scatter plot shows the increase of total YB-1 expression versus the expression of HA-tagged YB-1. A linear regression (*y* = *ax* + *b*) enables the estimation of the YB-1 overexpression level as well as the background of endogenous YB-1 level (*b*). Endogenous YB-1 levels were reduced about 4 times with siRNA (*b* = 0.033 for siRNA and 1.39 for siNeg). Similar results were obtained with YB-1 mutants (Supplementary Figure S8a). Scale bar: 40 μm. (**B**) Representative images showing the distribution of HA-tagged YB-1 mutants in HeLa cells pretreated with siRNA to decrease the endogenous expression of YB-1. Scale bar: 40 μm. (**C**) Representative images of SG assembly in HeLa cells expressing HA-Tagged YB-1 and pretreated with YB-1 siRNA. Arsenite (200 μM, 1 h) was used to trigger SG assembly in HeLa cells. Scale bar: 40 μm. (**D**) Number of SGs per cell versus HA-YB-1 expression level in HeLa cells pretreated with siRNA. Right panel: Window of expression level chosen to quantify SG assembly in cells expressing wild type and mutant YB-1. This window includes YB-1 expression level ranging from about 58% to 220% compared to endogenous YB-1 level (siNeg), according to the results presented in (A). (**E**) Violin plots of SG assembly in HeLa cells expressing indicated Ha-tagged proteins. Hela cells were pretreated with siRNA to decrease endogenous YB-1 level. The same window of HA expression level was selected for all the conditions, except for the control (empty plasmid). Fluorescence images were generated automatically with an HCS imager. SGs were detected automatically as described in Figure 6B. The median value is indicated for the number and area of SGs per cell. The total area of SG is the best indicator of SG assembly. **P < 0.01; *t*-test with two tails. The results of an independent experiment (duplicate) are shown in Supplementary Figure S8b.

We then investigated whether YB-1 overexpression can inhibit SG assembly independently of its RNA-unwinding activity in the cytoplasm. We used the two YB-1 mutants, YB-1-RK and -KY, displaying altered stem/loop destabilization activity *in vitro* as previously shown in our structural analysis (Figure 4A, B). The point mutations were introduced in the full length YB-1 protein for the experiments in cells. Endogenous YB-1 levels were also decreased to better capture the impact of the mutations in HeLa cells. HA-tagged wild-type and mutant YB-1 were therefore overexpressed in HeLa cells pretreated with siRNA targeting the 5′UTR of endogenous YB-1 transcripts. Under normal conditions, the spatial distribution of the YB-1 added back to HeLa cells, mutated or not, was not significantly affected, nor did we observe SGs (Figure 7A, B). The total level of YB-1 (WT + HA-tagged) versus the HA-tagged YB-1 level alone was then quantified at the single cell level for all the conditions tested (Figure 7A and Supplementary Figure S8a). To make a fair comparison, we then analyzed SG assembly in the same HA-tagged YB-1 expression window in the HeLa cells for wild type and mutant YB-1 (Figure 7D). When wild type YB-1 was added back to the HeLa cells with reduced levels of endogenous YB-1, we observed and measured a significant decrease in SG area per HeLa cell treated with arsenite (Figure 7C, E). However, the overexpression of YB-1C, YB-1-RK or -KY decreased the total SG area per HeLa cells to a lesser extent than wild type YB-1 (Figure 7C, E and Supplementary Figure S8b). Although a compensatory mechanism or other indirect mechanisms associated to YB-1 expression cannot be excluded, if we assume that mutating full length YB-1 would induce the same RNA-unwinding deficiency as observed for YB-1 C *in vitro*, the results obtained with YB-1-RK and -KY support a role for the RNA-unwinding activity of YB-1 in the negative regulation of SG assembly.

Interestingly, YB-1C with its short CTD (129–180, a.a.) has a reduced capacity to prevent SG assembly compared to full length YB-1 (1–324, a.a.). Thus, despite its self-adhesive properties *in vitro*, the long YB-1 CTD negatively contributes to SG assembly in the cells.

### Endogenous YB-1 promotes translation and SG dissociation in HeLa cells

**Figure 8.**
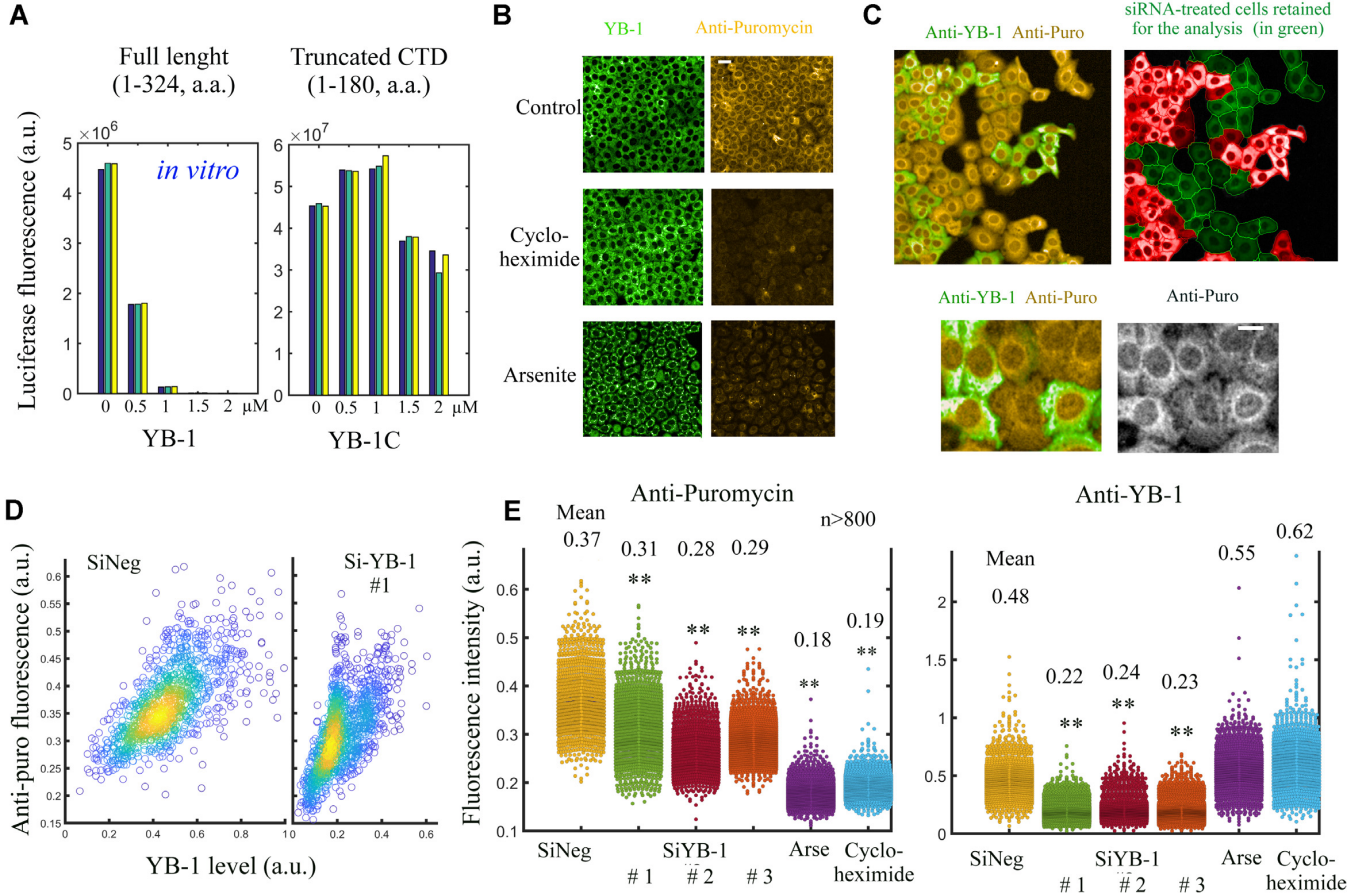
Endogenous YB-1 promotes global mRNA translation in Hela cells. (**A**) Rabbit reticulocyte translation assays in the presence of full length YB-1 or YB-1C. YB-1 was pre-mixed with Luciferase mRNA before translation took place. Each bar represents the results of a different experiment. Note the sharp decrease in translation with full length YB-1 but not YB-1C. Raw fluorescence data are shown in Supplementary Table S3. (**B**) Representative images of the incorporation of puromycin into nascent peptide chains in HeLa cells after a 10-min exposure to puromycin. When cells were treated with arsenite (200 μM) or cycloheximide (100 μg/ml) prior to puromycin exposure to stop translation, a significant decrease in puromycin incorporation was observed. Scale bar: 80 μm. (**C**) Upper left panel: HeLa cells were pretreated with siRNA-1 to decrease endogenous YB-1 expression and then briefly with puromycin to estimate global mRNA translation. In some areas, both YB-1 rich and -poor cells can be observed, which indicates an efficient decrease in endogenous YB-1 levels. Upper right panel: YB-1 poor cells were selected automatically for the analysis of the puromycin incorporation. Lower panel: Zoom in on representative cells displaying differential expression levels of endogenous YB-1 (anti-YB-1). Scale bar: 40 μm. (**D**) Puromycin incorporation level was measured at the single cell level in HeLa cells after siYB-1 or siNeg treatments. We noticed a moderate decrease of puromycin incorporation in siRNA-treated cells. (**E**) Scatter plots representing YB-1expression level and puromycin incorporation in HeLa cells exposed to indicated treatments. Three different siRNAs that significantly decrease YB-1 levels were used (Supplementary Figure S9a). We noticed a global decrease in anti-puromycin fluorescence in siRNA-pretreated cell compared to control cells (siNeg).

YB-1 is generally considered a negative regulator of mRNA translation. The compaction of mRNA into a beads-on-a-string structure by the YB-1 CTD *in vitro* (49) most likely prevents the scanning of 5′UTRs by translation preinitiation complexes (PICs). In agreement with this, YB-1, but not YB-1C, is a strong mRNA translation inhibitor *in vitro* (29) (Figure 8A). To probe the role of YB-1 in mRNA translation in HeLa cells, the translation rate was measured at the single cell level through the brief incorporation of puromycin into nascent peptide chains (58). As controls, cycloheximide, an inhibitor of translation elongation, and arsenite, which blocks translation initiation, dramatically decreased puromycin incorporation in HeLa cells (Figure 8B). In contrast with *in vitro* data showing negative translational regulation by YB-1, silencing YB-1 expression with three different siRNAs reduced overall puromycin incorporation in HeLa cells to about 16–24% (Figure 8C–E and Supplementary Figure S9a, short-term expression inhibition with siRNA has been preferred to reduce the risk of compensation mechanism). In support of this result, a positive regulation of polysome assembly by YB-1 has already been reported by others in Caenorhabditis elegans and myeloma cells (59,60). Although an mRNA-unwinding activity by YB-1 is the most direct explanation for the positive regulation of mRNA translation by YB-1, we cannot exclude an alternative interpretation such as the YB-1-dependent transcription or splicing regulation that would indirectly impact mRNA translation. To further support whether YB-1 mRNA-unwinding activity may positively regulate mRNA translation in HeLa cells, wild type or RNA-unwinding defective YB-1 mutants were added back to HeLa cells pretreated with siRNA to again decrease endogenous YB-1 expression. Globally, wild type or mutated YB-1 expression promoted mRNA translation in cells (Supplementary Figure S10). However, the RNA-unwinding defective mutants, YB-1-RK and-KY, were less efficient than wild type YB-1 in increasing translation, which suggests a possible contribution of the mRNA-unwinding activity mediated by YB-1. Finally, since puromycin incorporation is more pronounced for full length YB-1 than YB-1C, even if the difference is not significant under our experimental conditions, the long CTD does not appear to negatively regulate mRNA translation in cells.

**Figure 9.**
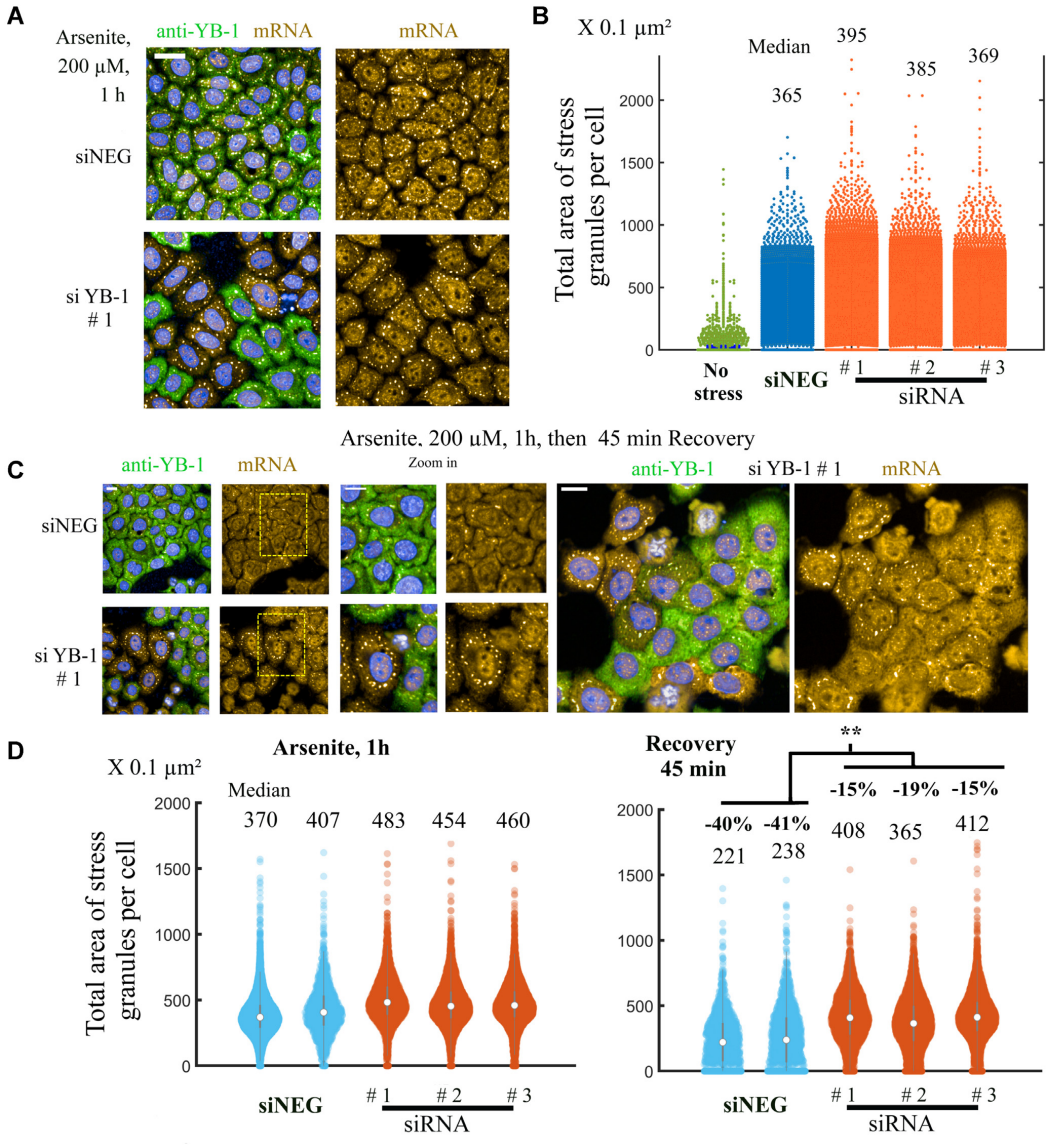
Endogenous YB-1 promotes SG disassembly during stress recovery. (**A**) HeLa cells were pretreated with siRNA-1 to decrease endogenous YB-1 expression or siNeg (control). HeLa cells were then exposed to arsenite (200 μM for 1 h), fixed and stained to observe the correlation between YB-1 levels (anti-YB-1 antibody) and SG assembly (in situ hybridization to detect mRNA). No significant difference in SG assembly was noticed between siRNA or siNeg treatment. Scale bar: 80 μm. (**B**) Scatter plot of the total area of SGs per cell measured after indicated treatments, as shown in Figure 6b. Three different siRNAs were used to decrease YB-1 levels (see Figure 7E for their validation). Median area values are indicated. (**C**) To initiate SG dissociation in HeLa, arsenite was washed out for 45 min. The different panels display representative imaged of SGs after indicated treatments. We noticed an increased occurrence of persistent SGs in HeLa cells displaying low YB-1 levels. Large scale images are shown in Supplementary Figure S9b for the two other siRNAs. Scale bars: 40 μm. (**D**) After indicated treatments, the total area of SG per cell was measured and plotted at the single cell level. SG disassembly is more pronounced in siNeg than siRNA-treated cells. **P < 0.01; *t*-test with two tails. %: decrease in median area values before and after recovery.

We then reconsidered whether endogenous YB-1 levels may negatively regulate SG assembly under physiological conditions. However, in agreement with previous reports (39,40) including a recent large scale CRISPR screen (42), decreasing the expression level of YB-1 by using three different siRNAs had no marked impact on SG assembly (Figure 9A, B). This result was expected as endogenous YB-1 levels are only sufficient to package non polysomal mRNAs, which represent a small fraction of total mRNAs in proliferating cells (1). Therefore, the number of YB-1 proteins may not be sufficient to prevent SG assembly after the massive dissociation of polysomes in arsenite-treated cells. Accordingly, we detected a slight increase in total area of SGs per cell after decreasing YB-1 levels (Figure 9B, D), which supports a negative regulation exerted by YB-1 on SG assembly. In search of a more pronounced phenotype, we then devised whether endogenous YB-1 levels may promote the progressive dissociation of SGs after stress recovery (61). Indeed, SGs are dynamical liquid-like condensates. Even after arsenite stress, SGs vanished with time. To estimate SG disassembly, the total area of SG per HeLa cell pretreated with arsenite before and after a 45-min recovery period was measured for the three different siRNAs directed against YB-1 expression (Figure 9C, D and Supplementary Figure S9b). In HeLa cells expressing endogenous YB-1, the dissociation of SGs after a 45-min recovery period was more significant (about 40%) than in HeLa cells treated with siRNAs (about 20%). These results clearly support a dissociation of SGs facilitated by endogenous YB-1 in HeLa cells.

## DISCUSSION

Protein synthesis requires the scanning of mRNAs by ribosomes, but, in cells, there are also dormant mRNAs waiting for the right place and the right time to be translated. Translation is also stochastic with active mRNAs remaining silent from time to time (62). Here, we provide the structural basis to understand how the association of YB-1 to non polysomal mRNAs contributes to keep them unstructured making them less prone to form liquid-like compartments and thus ready for translation.

### Why unwinding non polysomal mRNA is important, especially under stress conditions

Most mRNAs are continuously translated in proliferating cells, leaving only as small fraction of mRNA in a dormant state (1). Even if the fractions of polysomal and dormant mRNPs vary also significantly from transcript to transcript, abundant transcripts encoding housekeeping proteins are almost uninterruptedly translated in contrast with less abundant transcripts encoding regulatory factors such as transcription factors (1) for which translation is activated episodically.

It is known that mRNA-secondary structures in 5′UTRs can hinder translation but significantly less in CDS. Scanning ribosomes overcome these obstacles in CDS with a robust ATP-driven machinery that fuels helicases such as eIF4A. In agreement with this, large scale analysis of mRNA secondary structures in cells revealed significantly fewer secondary structures in coding sequences than expected (63–65). However, given the increase in the pool of repressed mRNA at the expense of polysomes under most stress conditions such as hypoxia and caloric restriction, the proper processing of repressed mRNA becomes an important challenge. The formation of strong secondary structures in dormant mRNPs may then prevent or delay translation recovery. RNA-binding proteins unwinding the secondary structure of repressed mRNA may therefore be important to maintain a basal level of translation, reprogram translation and facilitate the recovery after stress. If we consider the candidate proteins to limit mRNA secondary structures in dormant mRNPs, a specific attention has been given to YB-1, the major partner of non polysomal mRNAs along with PABP, which binds to the 3′end poly(A) tail (26,66).

### Structural basis for the mRNA-unwinding by YB-1

**Figure 10.**
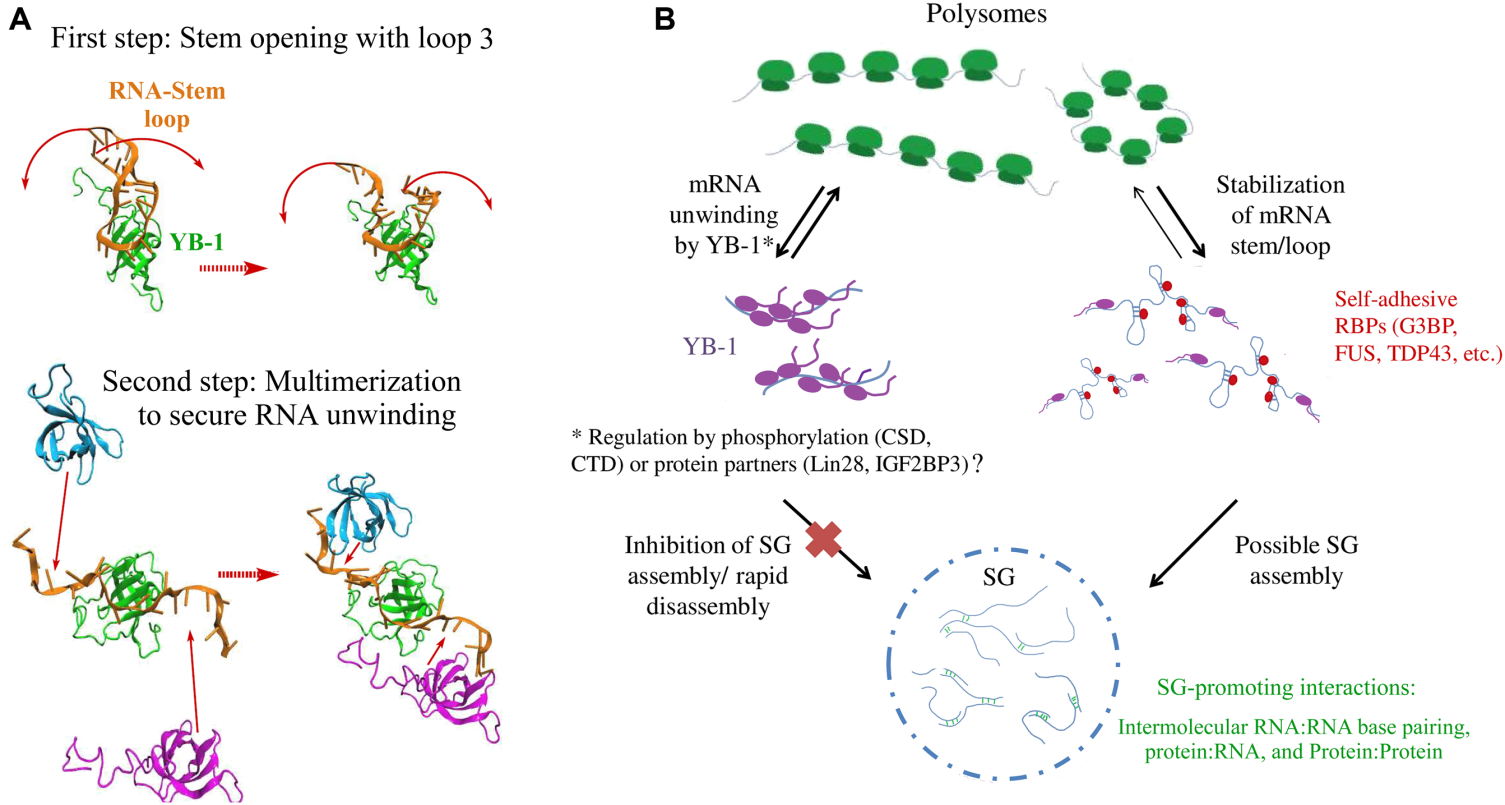
Mechanism behind YB-1 RNA-unwinding activity and putative underlying functions in cells. (**A**) View on the two transition pathways leading to an open RNA stem/loop. The upper figure illustrates the stem opening and the lower one illustrates the multimerization (Trimer here chosen arbitrarily). The RNA stem/loop and the YB-1 monomers are shown in cartoon and are colored in orange, cyan, green and magenta for the RNA and the monomers A, B and C, respectively. (**B**) Putative YB-1 functions in translation regulation through its association to non polysomal mRNA. By unwinding dormant mRNAs, YB-1 makes them ready to be activated for translation. Secondary structures in dormant mRNA delay the initiation of translation possibly by providing obstacles to the scanning of 5′ UTR by preinitiation complexes. RNA:RNA intermolecular interactions can also directly promote SG assembly. YB-1 protein partners and (or) phosphorylation events (S102, CTD) may have a critical role to negatively or positively regulate the RNA-unwinding activity of YB-1 *in vivo*.

YB-1 combines different properties that may play a key role in preventing the formation of mRNA secondary structures (10): a cold shock domain binding to mRNA with a weak specificity and a loop 3 longer and more positively charged compared with CSPs in bacteria or with Lin28 CSD (Supplementary Figure S4a). The structural analysis presented here shows a destabilization of RNA/stem loops by the charged residues located in CSD loop 3 and those located at the beginning of the C-terminal domain of YB-1C (1–180, a.a). In addition, we revealed an intramolecular interaction between CSD and CTD to orient the positively charged residues toward the stem (Figure 3A, C). Another interesting property of YB-1 relies on its ability to form a nucleoprotein filament in which consecutive YB-1 CSD domains are tightly packed along mRNA (12), leaving no room for secondary structures to regenerate (Figure 10A). The structural analysis also raises new questions. The long CSD loop 3 (a.a. 94–106) is also the target of AKT kinase, which possibly phosphorylates S102. As YB-1 phosphorylation may activate translation of dormant mRNA to increase cancer cell invasiveness and proliferation (27,67), it may be interesting to examine what is the impact of S102 phosphorylation on the unwinding activity exerted by YB-1. Phosphorylation also takes place in the positively charged CTD (68), which can explain the positive role of YB-1 in mRNA translation in HeLa cells while it efficiently stops translation *in vitro* and possibly in some tissues. In another scenario, the CTD residues (180–380 a.a.) are not involved in the binding to mRNA in cells. Instead, the self-adhesive interactions between CTDs would help to direct YB-1 on dormant mRNA and increase its capacity to unwind repressed mRNAs.

Interestingly, in contrast to helicases, the whole RNA-unwinding process does not require ATP, making it particularly relevant under stress conditions when the energy supply runs out.

### RNA-unwinding by YB-1 and SG assembly/disassembly

SG assembly is generated by either self-adhesive interactions between RNA-binding proteins such as G3BP-1(42) and intermolecular base pairing between mRNAs (7). Previous reports proposed a positive regulation of G3BP-1 expression by YB-1 in U2OS cells, which then promotes SG assembly (38). However, a correlation between the expression levels of YB-1 and G3BP-1 is not the norm (39). In HeLa cells, YB-1 is a negative regulator of SG assembly with few SGs assembled in YB-1-overexpressing cells (Figure 6A, B), in contrast with many other RBPs but similar to eIF4A or DDX19A (9) and possibly IF2GBP3 (Supplementary Figure S6a,b). As eIF4A and DDX19A are RNA helicases that can prevent SG assembly, a role for the RNA-unwinding activity of YB-1 in controlling SG assembly by inhibiting the formation of mRNA intermolecular bridges makes sense. Moreover, we identified two RNA-unwinding defective YB-1C mutants *in vitro* (Figure 4). The overexpression of full length YB-1 mutants prevented SG assembly less efficiently than wild type YB-1 (Figure 7E). Finally, even without YB-1 overexpression, endogenous YB-1 levels clearly promoted SG disassembly during arsenite stress recovery in HeLa cells (Figure 9C, D).

With its long and self-adhesive CTD, YB-1 may have been considered a positive regulator of SG assembly. *In vitro*, the YB-1 CTD indeed favors the compaction of mRNA, prevents translation and promotes YB-1 self-attraction, which is the reason why full length YB-1 is not yet amenable to NMR spectroscopy. However full length YB-1 is more potent in preventing SG assembly in HeLa cells than YB-1C with its short CTD. Whether the YB-1 CTD facilitates RNA unwinding remains to be demonstrated as well, as does a role for the multiple phosphorylation sites. The YB-1 CTD may simply increase the affinity of YB-1 for mRNAs to promote the RNA chaperone activity of YB-1. An attractive hypothesis is that YB-1 cellular functions related to mRNA can be regulated in cell type-dependent and contextual manner. On the one hand, YB-1 unwinds mRNA, inhibits SG assembly and promotes translation in cancer cells, as shown in this study (Figure 10B). On the other hand, YB-1 preserves RNA secondary structure, compacts mRNAs to maintain their dormancy, and positively regulates the formation of mRNA-rich condensates in some tissues. Precise spatio-temporal regulation of YB-1 mRNP activation can possibly be orchestrated by posttranslational events in the CTD and CSD (27,34,67) and protein partners (36,54).

The discovery of the molecular mechanisms by which YB-1 packages dormant mRNPs will increase our understanding of how mammalian cells respond to stress at the translational level under various stimuli such as caloric restriction in rats, which upregulates YB-1 expression (46). Cellular plasticity in gene expression notably participates in cancer cell resistance under relevant conditions in affected humans (oxidative stress, hypoxia, and chemotherapy). Although the translation of new genes can be undertaken from newly transcribed mRNAs, the most rapid response to stress relies on the translation of dormant transcripts that can be rapidly activated. Altogether the results presented here enlighten the molecular mechanism behind the YB-1 RNA-unwinding activity which may help cells cope with stress, notably by facilitating SG disassembly and subsequent de novo translation in cells.

## DATA AVAILABILITY

All the data presented in this study are available on request from the corresponding author.

## Supplementary Material

gkab748_Supplemental_Files

## References

[1] Hendrickson D.G., Hogan D.J., McCullough H.L., Myers J.W., Herschlag D., Ferrell J.E., Brown P.O. Concordant regulation of translation and mRNA abundance for hundreds of targets of a human microRNA. PLoS Biol. 2009; 7:e1000238. 19901979 10.1371/journal.pbio.1000238 PMC2766070

[2] Wek R.C., Jiang H.-Y., Anthony T.G. Coping with stress: eIF2 kinases and translational control. Biochem. Soc. Trans. 2006; 34:7–11. 16246168 10.1042/BST20060007

[3] Ivanov P., Kedersha N., Anderson P. Stress granules and processing bodies in translational control. Cold Spring Harb. Perspect. Biol. 2019; 11:a032813. 30082464 10.1101/cshperspect.a032813 PMC6496347

[4] Yang P., Mathieu C., Kolaitis R.-M., Zhang P., Messing J., Yurtsever U., Yang Z., Wu J., Li Y., Pan Q. G3BP1 is a tunable switch that triggers phase separation to assemble stress granules. Cell. 2020; 181:325–345. 32302571 10.1016/j.cell.2020.03.046 PMC7448383

[5] Sanders D.W., Kedersha N., Lee D.S., Strom A.R., Drake V., Riback J.A., Bracha D., Eeftens J.M., Iwanicki A., Wang A. Competing protein-RNA interaction networks control multiphase intracellular organization. Cell. 2020; 181:306–324. 32302570 10.1016/j.cell.2020.03.050 PMC7816278

[6] Guillén-Boixet J., Kopach A., Holehouse A.S., Wittmann S., Jahnel M., Schlüßler R., Kim K., Trussina I.R., Wang J., Mateju D. RNA-induced conformational switching and clustering of G3BP drive stress granule assembly by condensation. Cell. 2020; 181:346–361. 32302572 10.1016/j.cell.2020.03.049 PMC7181197

[7] Van Treeck B., Protter D.S., Matheny T., Khong A., Link C.D., Parker R. RNA self-assembly contributes to stress granule formation and defining the stress granule transcriptome. Proc. Natl. Acad. Sci. U.S.A. 2018; 115:2734–2739. 29483269 10.1073/pnas.1800038115 PMC5856561

[8] De Groot N.S., Armaos A., Graña-Montes R., Alriquet M., Calloni G., Vabulas R.M., Tartaglia G.G. RNA structure drives interaction with proteins. Nat. Commun. 2019; 10:3246. 31324771 10.1038/s41467-019-10923-5 PMC6642211

[9] Tauber D., Tauber G., Khong A., Van Treeck B., Pelletier J., Parker R. Modulation of RNA condensation by the DEAD-box protein eIF4A. Cell. 2020; 180:411–426. 31928844 10.1016/j.cell.2019.12.031 PMC7194247

[10] Lyabin D.N., Eliseeva I.A., Ovchinnikov L.P. YB-1 protein: functions and regulation. Wiley Interdiscipl. Rev.: RNA. 2014; 5:95–110. 10.1002/wrna.1200 24217978

[11] Kohno K., Izumi H., Uchiumi T., Ashizuka M., Kuwano M. The pleiotropic functions of the Y-box-binding protein, YB-1. Bioessays. 2003; 25:691–698. 12815724 10.1002/bies.10300

[12] Kretov D.A., Clément M.-J., Lambert G., Durand D., Lyabin D.N., Bollot G., Bauvais C., Samsonova A., Budkina K., Maroun R.C. YB-1, an abundant core mRNA-binding protein, has the capacity to form an RNA nucleoprotein filament: a structural analysis. Nucleic Acids Res. 2019; 47:3127–3141. 30605522 10.1093/nar/gky1303 PMC6451097

[13] Skabkin M.A., Evdokimova V., Thomas A.A., Ovchinnikov L.P. The major messenger ribonucleoprotein particle protein p50 (YB-1) promotes nucleic acid strand annealing. J. Biol. Chem. 2001; 276:44841–44847. 11585833 10.1074/jbc.M107581200

[14] Lindquist J.A., Mertens P.R. Cold shock proteins: from cellular mechanisms to pathophysiology and disease. Cell. Commun. Signal. 2018; 16:63. 30257675 10.1186/s12964-018-0274-6 PMC6158828

[15] Zhang Y., Burkhardt D.H., Rouskin S., Li G.-W., Weissman J.S., Gross C.A. A stress response that monitors and regulates mRNA structure is central to cold shock adaptation. Mol. Cell. 2018; 70:274–286. 29628307 10.1016/j.molcel.2018.02.035 PMC5910227

[16] Lu Z.H., Books J.T., Ley T.J. YB-1 is important for late-stage embryonic development, optimal cellular stress responses, and the prevention of premature senescence. Mol. Cell Biol. 2005; 25:4625. 15899865 10.1128/MCB.25.11.4625-4637.2005 PMC1140647

[17] Jayavelu A.K., Schnöder T.M., Perner F., Herzog C., Meiler A., Krishnamoorthy G., Huber N., Mohr J., Edelmann-Stephan B., Austin R. Splicing factor YBX1 mediates persistence of JAK2-mutated neoplasms. Nature. 2020; 588:157–163. 33239784 10.1038/s41586-020-2968-3

[18] Bargou R.C., Jürchott K., Wagener C., Bergmann S., Metzner S., Bommert K., Mapara M.Y., Winzer K.-J., Dietel M., Dörken B. Nuclear localization and increased levels of transcription factor YB-1 in primary human breast cancers are associated with intrinsic MDR1 gene expression. Nat. Med. 1997; 3:447–450. 9095180 10.1038/nm0497-447

[19] Li D., Liu X., Zhou J., Hu J., Zhang D., Liu J., Qiao Y., Zhan Q. Long noncoding RNA HULC modulates the phosphorylation of YB-1 through serving as a scaffold of extracellular signal–regulated kinase and YB-1 to enhance hepatocarcinogenesis. Hepatology. 2017; 65:1612–1627. 28027578 10.1002/hep.29010

[20] Zhang E., He X., Zhang C., Su J., Lu X., Si X., Chen J., Yin D., Han L., De W. A novel long noncoding RNA HOXC-AS3 mediates tumorigenesis of gastric cancer by binding to YBX1. Genome Biol. 2018; 19:154. 30286788 10.1186/s13059-018-1523-0 PMC6172843

[21] Gandhi M., Groß M., Holler J.M., Si’Ana A.C., Patil N., Leupold J.H., Munschauer M., Schenone M., Hartigan C.R., Allgayer H. The lncRNA lincNMR regulates nucleotide metabolism via a YBX1-RRM2 axis in cancer. Nat. Commun. 2020; 11:3214. 32587247 10.1038/s41467-020-17007-9 PMC7316977

[22] Zhang E., He X., Zhang C., Su J., Lu X., Si X., Chen J., Yin D., Han L., De W. A novel long noncoding RNA HOXC-AS3 mediates tumorigenesis of gastric cancer by binding to YBX1. Genome Biol. 2018; 19:154. 30286788 10.1186/s13059-018-1523-0 PMC6172843

[23] de Souza-Pinto N.C., Mason P.A., Hashiguchi K., Weissman L., Tian J., Guay D., Lebel M., Stevnsner T.V., Rasmussen L.J., Bohr V.A. Novel DNA mismatch-repair activity involving YB-1 in human mitochondria. DNA Repair (Amst.). 2009; 8:704–719. 19272840 10.1016/j.dnarep.2009.01.021 PMC2693314

[24] Alemasova E.E., Naumenko K.N., Kurgina T.A., Anarbaev R.O., Lavrik O.I. The multifunctional protein YB-1 potentiates PARP1 activity and decreases the efficiency of PARP1 inhibitors. Oncotarget. 2018; 9:23349. 29805738 10.18632/oncotarget.25158 PMC5955111

[25] Gopal S.K., Greening D.W., Mathias R.A., Ji H., Rai A., Chen M., Zhu H.-J., Simpson R.J. YBX1/YB-1 induces partial EMT and tumourigenicity through secretion of angiogenic factors into the extracellular microenvironment. Oncotarget. 2015; 6:13718. 25980435 10.18632/oncotarget.3764 PMC4537044

[26] Singh G., Pratt G., Yeo G.W., Moore M.J. The clothes make the mRNA: past and present trends in mRNP fashion. Annu. Rev. Biochem. 2015; 84:325–354. 25784054 10.1146/annurev-biochem-080111-092106 PMC4804868

[27] Evdokimova V., Ruzanov P., Anglesio M.S., Sorokin A.V., Ovchinnikov L.P., Buckley J., Triche T.J., Sonenberg N., Sorensen P.H. Akt-mediated YB-1 phosphorylation activates translation of silent mRNA species. Mol. Cell Biol. 2006; 26:277–292. 16354698 10.1128/MCB.26.1.277-292.2006 PMC1317623

[28] Wu S.-L., Fu X., Huang J., Jia T.-T., Zong F.-Y., Mu S.-R., Zhu H., Yan Y., Qiu S., Wu Q. Genome-wide analysis of YB-1-RNA interactions reveals a novel role of YB-1 in miRNA processing in glioblastoma multiforme. Nucleic Acids Res. 2015; 43:8516–8528. 26240386 10.1093/nar/gkv779 PMC4787835

[29] Nekrasov M.P., Ivshina M.P., Chernov K.G., Kovrigina E.A., Evdokimova V.M., Thomas A.A., Hershey J.W., Ovchinnikov L.P. The mRNA-binding protein YB-1 (p50) prevents association of the eukaryotic initiation factor eIF4G with mRNA and inhibits protein synthesis at the initiation stage. J. Biol. Chem. 2003; 278:13936–13943. 12582179 10.1074/jbc.M209145200

[30] Evdokimova V., Tognon C., Ng T., Ruzanov P., Melnyk N., Fink D., Sorokin A., Ovchinnikov L.P., Davicioni E., Triche T.J. Translational activation of snail1 and other developmentally regulated transcription factors by YB-1 promotes an epithelial-mesenchymal transition. Cancer Cell. 2009; 15:402–415. 19411069 10.1016/j.ccr.2009.03.017

[31] El-Naggar A.M., Veinotte C.J., Cheng H., Grunewald T.G., Negri G.L., Somasekharan S.P., Corkery D.P., Tirode F., Mathers J., Khan D. Translational activation of HIF1α by YB-1 promotes sarcoma metastasis. Cancer Cell. 2015; 27:682–697. 25965573 10.1016/j.ccell.2015.04.003

[32] Uchiumi T., Fotovati A., Sasaguri T., Shibahara K., Shimada T., Fukuda T., Nakamura T., Izumi H., Tsuzuki T., Kuwano M. YB-1 is important for an early stage embryonic development: neural tube formation and cell proliferation. J. Biol. Chem. 2006; 281:40440–40449. 17082189 10.1074/jbc.M605948200

[33] Lim J.P., Shyamasundar S., Gunaratne J., Scully O.J., Matsumoto K., Bay B.H. YBX1 gene silencing inhibits migratory and invasive potential via CORO1C in breast cancer in vitro. BMC Cancer. 2017; 17:201. 28302118 10.1186/s12885-017-3187-7 PMC5356414

[34] El-Naggar A.M., Somasekharan S.P., Wang Y., Cheng H., Negri G.L., Pan M., Wang X.Q., Delaidelli A., Rafn B., Cran J. Class I HDAC inhibitors enhance YB-1 acetylation and oxidative stress to block sarcoma metastasis. EMBO Rep. 2019; 20:e48375. 31668005 10.15252/embr.201948375 PMC6893361

[35] Bader A., Vogt P. Phosphorylation by Akt disables the anti-oncogenic activity of YB-1. Oncogene. 2008; 27:1179–1182. 17704806 10.1038/sj.onc.1210719

[36] Samsonova A., El Hage K., Desforges B., Joshi V., Clément M.-J., Lambert G., Henrie H., Babault N., Craveur P., Maroun R.C. Lin28, a major translation reprogramming factor, gains access to YB-1-packaged mRNA through its cold-shock domain. Commun. Biol. 2021; 4:359. 33742080 10.1038/s42003-021-01862-3 PMC7979924

[37] Tafuri S.R., Familari M., Wolffe A.P. A mouse Y box protein, MSY1, is associated with paternal mRNA in spermatocytes. J. Biol. Chem. 1993; 268:12213–12220. 8505341

[38] Somasekharan S.P., El-Naggar A., Leprivier G., Cheng H., Hajee S., Grunewald T.G., Zhang F., Ng T., Delattre O., Evdokimova V. YB-1 regulates stress granule formation and tumor progression by translationally activating G3BP1. J. Cell Biol. 2015; 208:913–929. 25800057 10.1083/jcb.201411047 PMC4384734

[39] Lyons S.M., Achorn C., Kedersha N.L., Anderson P.J., Ivanov P. YB-1 regulates tiRNA-induced Stress Granule formation but not translational repression. Nucleic Acids Res. 2016; 44:6949–6960. 27174937 10.1093/nar/gkw418 PMC5001593

[40] Bounedjah O., Desforges B., Wu T.-D., Pioche-Durieu C., Marco S., Hamon L., Curmi P.A., Guerquin-Kern J.-L., Piétrement O., Pastré D. Free mRNA in excess upon polysome dissociation is a scaffold for protein multimerization to form stress granules. Nucleic Acids Res. 2014; 42:8678–8691. 25013173 10.1093/nar/gku582 PMC4117795

[41] Bley N., Lederer M., Pfalz B., Reinke C., Fuchs T., Glaß M., Möller B., Hüttelmaier S. Stress granules are dispensable for mRNA stabilization during cellular stress. Nucleic Acids Res. 2015; 43:e26. 25488811 10.1093/nar/gku1275 PMC4344486

[42] Wheeler E.C., Vu A.Q., Einstein J.M., DiSalvo M., Ahmed N., Van Nostrand E.L., Shishkin A.A., Jin W., Allbritton N.L., Yeo G.W. Pooled CRISPR screens with imaging on microraft arrays reveals stress granule-regulatory factors. Nat. Methods. 2020; 17:636–642. 32393832 10.1038/s41592-020-0826-8 PMC7357298

[43] Tanaka T., Ohashi S., Kobayashi S. Roles of YB-1 under arsenite-induced stress: translational activation of HSP70 mRNA and control of the number of stress granules. Biochim. Biophys. Acta (BBA)-Gen. Subj. 2014; 1840:985–992. 10.1016/j.bbagen.2013.11.002 24231679

[44] Anderson P., Kedersha N. Visibly stressed: the role of eIF2, TIA-1, and stress granules in protein translation. Cell Stress Chaperones. 2002; 7:213. 12380690 10.1379/1466-1268(2002)007<0213:vstroe>2.0.co;2 PMC514820

[45] Colombrita C., Zennaro E., Fallini C., Weber M., Sommacal A., Buratti E., Silani V., Ratti A. TDP-43 is recruited to stress granules in conditions of oxidative insult. J. Neurochem. 2009; 111:1051–1061. 19765185 10.1111/j.1471-4159.2009.06383.x

[46] Ma S., Sun S., Geng L., Song M., Wang W., Ye Y., Ji Q., Zou Z., Wang S., He X. et al. Caloric restriction reprograms the Single-Cell transcriptional landscape of rattus norvegicus aging. Cell. 2020; 180:984–1001. 32109414 10.1016/j.cell.2020.02.008

[47] Wang L., Nam Y., Lee A.K., Yu C., Roth K., Chen C., Ransey E.M., Sliz P. LIN28 zinc knuckle domain is required and sufficient to induce let-7 oligouridylation. Cell Rep. 2017; 18:2664–2675. 28297670 10.1016/j.celrep.2017.02.044 PMC13247719

[48] Kloks C.P., Spronk C.A., Lasonder E., Hoffmann A., Vuister G.W., Grzesiek S., Hilbers C.W. The solution structure and DNA-binding properties of the cold-shock domain of the human Y-box protein YB-1. J. Mol. Biol. 2002; 316:317–326. 11851341 10.1006/jmbi.2001.5334

[49] Skabkin M.A., Kiselyova O.I., Chernov K.G., Sorokin A.V., Dubrovin E.V., Yaminsky I.V., Vasiliev V.D., Ovchinnikov L.P. Structural organization of mRNA complexes with major core mRNP protein YB-1. Nucleic Acids Res. 2004; 32:5621–5635. 15494450 10.1093/nar/gkh889 PMC524299

[50] Evdokimova V.M., Wei C.-L., Sitikov A.S., Simonenko P.N., Lazarev O.A., Vasilenko K.S., Ustinov V.A., Hershey J.W., Ovchinnikov L.P. The major protein of messenger ribonucleoprotein particles in somatic cells is a member of the Y-box binding transcription factor family. J. Biol. Chem. 1995; 270:3186–3192. 7852402 10.1074/jbc.270.7.3186

[51] Gaudreault I., Guay D., Lebel M. YB-1 promotes strand separation in vitro of duplex DNA containing either mispaired bases or cisplatin modifications, exhibits endonucleolytic activities and binds several DNA repair proteins. Nucleic Acids Res. 2004; 32:316–327. 14718551 10.1093/nar/gkh170 PMC373280

[52] Kretov D.A., Curmi P.A., Hamon L., Abrakhi S., Desforges B., Ovchinnikov L.P., Pastré D. mRNA and DNA selection via protein multimerization: YB-1 as a case study. Nucleic Acids Res. 2015; 43:9457–9473. 26271991 10.1093/nar/gkv822 PMC4627072

[53] Zhang J., Fan J.-S., Li S., Yang Y., Sun P., Zhu Q., Wang J., Jiang B., Yang D., Liu M. Structural basis of DNA binding to human YB-1 cold shock domain regulated by phosphorylation. Nucleic Acids Res. 2020; 48:9361–9371. 32710623 10.1093/nar/gkaa619 PMC7498358

[54] Feng M., Xie X., Han G., Zhang T., Li Y., Li Y., Yin R., Wang Q., Zhang T., Wang P. YBX1 is required for maintaining myeloid leukemia cell survival by regulating BCL2 stability in an m6A-dependent manner. Blood. 2021; 138:71–85. 33763698 10.1182/blood.2020009676 PMC8667054

[55] Nagaraj N., Wisniewski J.R., Geiger T., Cox J., Kircher M., Kelso J., Pääbo S., Mann M. Deep proteome and transcriptome mapping of a human cancer cell line. Mol. Syst. Biol. 2011; 7:548. 22068331 10.1038/msb.2011.81 PMC3261714

[56] Lai A., Valdez-Sinon A.N., Bassell G.J. Regulation of RNA granules by FMRP and implications for neurological diseases. Traffic. 2020; 21:454–462. 32374065 10.1111/tra.12733 PMC7377269

[57] Abraham M.J., Murtola T., Schulz R., Páll S., Smith J.C., Hess B., Lindahl E. GROMACS: high performance molecular simulations through multi-level parallelism from laptops to supercomputers. SoftwareX. 2015; 1:19–25.

[58] Schmidt E.K., Clavarino G., Ceppi M., Pierre P. SUnSET, a nonradioactive method to monitor protein synthesis. Nat. Methods. 2009; 6:275–277. 19305406 10.1038/nmeth.1314

[59] Bommert K.S., Effenberger M., Leich E., Kuspert M., Murphy D., Langer C., Moll R., Janz S., Mottok A., Weissbach S. et al. The feed-forward loop between YB-1 and MYC is essential for multiple myeloma cell survival. Leukemia. 2013; 27:441–450. 22772059 10.1038/leu.2012.185 PMC4047128

[60] Arnold A., Rahman M.M., Lee M.C., Muehlhaeusser S., Katic I., Gaidatzis D., Hess D., Scheckel C., Wright J.E., Stetak A. et al. Functional characterization of C. elegans Y-box-binding proteins reveals tissue-specific functions and a critical role in the formation of polysomes. Nucleic Acids Res. 2014; 42:13353–13369. 25378320 10.1093/nar/gku1077 PMC4245946

[61] Wheeler J.R., Matheny T., Jain S., Abrisch R., Parker R. Distinct stages in stress granule assembly and disassembly. Elife. 2016; 5:e18413. 27602576 10.7554/eLife.18413 PMC5014549

[62] Yan X., Hoek T.A., Vale R.D., Tanenbaum M.E. Dynamics of translation of single mRNA molecules in vivo. Cell. 2016; 165:976–989. 27153498 10.1016/j.cell.2016.04.034 PMC4889334

[63] Rouskin S., Zubradt M., Washietl S., Kellis M., Weissman J.S. Genome-wide probing of RNA structure reveals active unfolding of mRNA structures in vivo. Nature. 2014; 505:701–705. 24336214 10.1038/nature12894 PMC3966492

[64] Beaudoin J.-D., Novoa E.M., Vejnar C.E., Yartseva V., Takacs C.M., Kellis M., Giraldez A.J. Analyses of mRNA structure dynamics identify embryonic gene regulatory programs. Nat. Struct. Mol. Biol. 2018; 25:677–686. 30061596 10.1038/s41594-018-0091-z PMC6690192

[65] Sun L., Fazal F.M., Li P., Broughton J.P., Lee B., Tang L., Huang W., Kool E.T., Chang H.Y., Zhang Q.C. RNA structure maps across mammalian cellular compartments. Nat. Struct. Mol. Biol. 2019; 26:322–330. 30886404 10.1038/s41594-019-0200-7 PMC6640855

[66] Kumar A., Pederson T. Comparison of proteins bound to heterogeneous nuclear RNA and messenger RNA in HeLa cells. J. Mol. Biol. 1975; 96:353–365. 1165583 10.1016/0022-2836(75)90165-5

[67] Kosnopfel C., Sinnberg T., Sauer B., Busch C., Niessner H., Schmitt A., Forchhammer S., Grimmel C., Mertens P.R., Hailfinger S. YB-1 expression and phosphorylation regulate tumorigenicity and invasiveness in melanoma by influencing EMT. Mol. Cancer Res. 2018; 16:1149–1160. 29743296 10.1158/1541-7786.MCR-17-0528

[68] Sharma K., D'Souza R.C., Tyanova S., Schaab C., Wisniewski J.R., Cox J., Mann M. Ultradeep human phosphoproteome reveals a distinct regulatory nature of Tyr and Ser/Thr-based signaling. Cell Rep. 2014; 8:1583–1594. 25159151 10.1016/j.celrep.2014.07.036

