# Supplementary material for: YB-1 unwinds mRNA secondary structures in vitro and negatively regulates stress granule assembly in HeLa cells
Source: Nucleic Acids Res. 2021 Sep 1;49(17):10061–81. doi: 10.1093/nar/gkab748 (PMC8464072; doi:10.1093/nar/gkab748)
Supplement: gkab748_Supplemental_Files [file gkab748_supplemental_files.zip › All supplementary tables.pdf]

Table S1. Interaction energy between the two RNA strands of the stem and between individual base pairs along the stem, for the WT YB-1 and the two double mutants R97A-K98A and K137A-Y138A. Energies were averaged over the whole MD trajectory (200 ns).

**ΔE (kj/mol)**	**WT**	**R97A-K98A**	**K137A-Y138A**
**RNA-stem**	**-11851.7**	**-12121.9**	**-12096.7**
RNA base pairs			
A1-U24	-1.5	-9.7	-19.9
C2-G23	-57.7	-86.4	-73.2
A3-U22	-45.1	-21.8	-43.9
G4-C21	-80.5	-85.9	-85.7
A5-U20	-11.0	-38.3	-33.1
C6-G19	-66.0	-87.9	-85.1
A7-U18	-41.9	-44.5	-44.3
G8-C17	-85.0	-87.6	-86.9
A9-U16	-28.3	-34.3	-0.7
**Total RNA base pairs**	**-417.1**	**-496.3**	**-472.8**

Table S2. Interaction energy between RNA and different regions of YB-1. Energies were calculated for the WT complex and the two double mutants R97A-K98A and K137A-Y138A. Energies were averaged over the whole MD trajectory (200 ns).

**ΔE (kj/mol)**	**WT**	**R97A-K98A**	**K137A-Y138A**
RNA/YB-1	-1568.1	-1164.4	-1395.0
RNA/CSD	-1113.1	-833.4	-931.6
RNA/β-loop	*-373.3*	*-62.0*	*-314.7*
RNA/C-ter	-455.6	-331.8	-464.0
β-loop/C-ter	-284.9	-355.9	-332.7
RNA/R97	*-183.7*	*-1.5*	*-117.9*
RNA/K98	*-95.1*	*-2.3*	*-21.1*
RNA/K137	-0.1	0.0	-0.1
RNA/Y138	-0.2	-0.2	-0.2

Table S3

Luciferase activity after translation in rabbit reticulocyte lysate YB-1 full length

YB-1 concentration	Repeats			Mean
	1	2	3	
0	4471895	4598785	4589165	4553281.7
0,5 µM	1780611	1784120	1805650	1790127
1 µM	131317	136419	141256	136330.67
1,5 µM	11793	12641	13985	12806.333
2 µM	3855	3877	3489	3740.3333

Luciferase activity after translation in rabbit reticulocyte lysate YB-1C (1-180)

YB-1C concentration	Repeats			Mean
	1	2	3	
0	45342612	45898521	45278956	45506696
0,5 µM	53934521	53774156	53621484	53776720
1 µM	54218113	54840812	57358572	55472499
1,5 µM	36940230	37985400	37895421	37607017
2 µM	34539129	29296984	33587920	32474678
