# Supplementary material for: YB-1 unwinds mRNA secondary structures in vitro and negatively regulates stress granule assembly in HeLa cells
Source: Nucleic Acids Res. 2021 Sep 1;49(17):10061–81. doi: 10.1093/nar/gkab748 (PMC8464072; doi:10.1093/nar/gkab748)
Supplement: gkab748_Supplemental_Files [file gkab748_supplemental_files.zip › SUPPLEMENTARY FIGURE CAPTIONS.docx]

**SUPPLEMENTARY FIGURE CAPTIONS:**

**Figure S1: Analysis of the imino protons of a DNA stem/loop in the presence of YB-1 by NMR**

1. Upper left panel: 1D 1H NMR spectra of non-exchangeable protons of the ACT-4G stem/loop (ACAGACAGAACCCCTTCTGTCTGT, Tm ~ 60°C) for indicated YB-1C:DNA molar ratio. Right panel: Peak intensity variations of 20 non exchangeable protons. Lower left panel: Mean value of the bound:free intensity ratio at increasing concentrations of YB-1C. NMR spectra were recorded at 25°C with 50 µM ACT-4G DNA.
2. Left panel: 1D 1H NMR spectra of imino protons (specific to base pairing) of the ACT-4G stem/loop for indicated YB-1C:DNA molar ratio. Upper right panel: Intensity ratio variations of 6 imino protons. Lower right panel: Mean value of the bound:free intensity ratio at increasing YB-1C:DNA molar ratio. We noticed that the decrease in peak intensities with YB-1C concentrations is steeper for imino than non-exchangeable protons.

**Figure S2: YB-1 destabilizes DNA stem/loops but not its CSD fragment alone nor the two RRMs of TDP-43.**

1. Two-dimensional ^1^H-^15^N SOFAST-HSQC spectra of YB-1C (1-180aa.), the CSD alone (50-129) and the two RRMs of TDP-43 (101-269) in the presence of DNA stem/loop ((A)_10_-CCCC-(T)_10_), an unstructured DNA mimicking on open stem/loop ((A)_10_-CCCC-(A)_10_)) or a 30-nt long poly(dC) oligonucleotide (for YB-1C).
2. Intensity ratios after the addition of DNA stem/loop for YB-1C. Note the general decrease in peak heights in the structured CSD domain (50-129) due to its multimerization along ssDNA(24). *, multiple peaks. Multiple peaks are notably observed in the cold shock domain that interacts with ssDNA.
3. Left panel: In contrast with YB-1C, the RRMs of TDP-43 interacts with stem/loop without displaying multiple peaks. We also observe no significant decrease in peak heights in the presence of DNA stem/loop. Right panel: TDP-43 RRM structure (Lukavsky et al, 2013, NSMB), in interaction with unstructured GU repeats showing the residues represented in the left panel.

**Figure S3: NMR analysis reveals an RNA-unwinding activity dependent on an interaction between CSD loop 3 and CTD residues.**

1. CSP analysis from two-dimensional 1H-15N SOFAST-HSQC spectra of YB-1C (1-180, a.a.) in the presence a DNA stem/loop ((A)_10_-CCCC-(T)_10_) and an unstructured ssDNA that mimics the opening of the stem/loop ((A)_10_-CCCC-(A)_10_). We noticed a similar CSP signature. However, when the CSD alone interacts with the same ssDNA, marked differences appear in residues W65, F66, N70, G71 G73 and N94, which indicates a different binding of CSD to the stem/loop and unstructured ssDNA. *, invisible peaks.
2. Two-dimensional 1H-15N HSQC spectra of free wild type and mutant YB-1C.
3. CSPs of indicated YB-1 mutants versus wild type YB-1. Residues around the loop and in the CTD display similar CSPs for YB-1-RK and -KY.
4. Electrophoretic mobility of mRNA in the presence of wild type and mutant YB-1 (1-180, a.a.).

**Figure S4: The long and positively charged CSD loop 3, the beginning of the CTD and the first cluster of CTD arginine residues are specific to YB-1 and highly conserved.**

1. YB-1, but not Lin28 and bacterial cold-shock proteins, has a long CSD loop 3 that harbors additional positively charge residues not found in Lin28a, a cold-shock protein that has a high sequence similarity with YB-1. Additional K or R residues (in blue). CSD loop 3 in grey. Yellow: KKNNRKYL motif (92-100, a.a.) found only in YB-1 just before R101/S102 that is conserved in Lin28 (in red).
2. Conservations of the charged and long CSD loop 3 (in gray), the putative CTD residues interacting with CSD loop 3 (in red), and CTD arginine residues (in blue) across species

**Figure S5: YB-1-RK and KY have an impaired capacity to destabilize RNA stem/loop**

1. Structures of YB-KY and YB-KY with stem/loop RNA sampled from 200 ns of MD simulation showing the evolution in time of the interaction starting from t=0 ns (left), 100 ns (middle) and 200 ns (right). Residues interacting with RNA are colored in magenta. The four residues considered for mutation (R97, K98, K137 and Y138) are colored in black. RNA is colored in orange and the protein in green.
2. Hydrogen bonds formed between the arginine and lysine residues of β-loop and C-ter and the nucleic acids of the stem throughout the MD trajectory. Hydrogen (H) bonds were counted between donors (D) and acceptors (A) provided that the D-A distance is less than 3.0 Å and the D-H-A angle is less than 20 degrees. The upper (green) and lower (blue) panels are for the, R97A/K98A and K138A/Y137A complexes.
3. Structures of wild type YB-1 with stem/loop RNA sampled from 200 ns of MD simulation showing specific interactions between RK and RNA and residues from the β-loop and C-ter. RNA is colored in orange and the protein in green.
4. Evolution of the RMSD of different YB-1 domains during 200 ns of MD simulations for wild type YB-1 and indicated mutants. The RMSD from the starting conformation of WT YB-1 was calculated on the Cα atoms for: the whole protein (top panel), the C-terminal segment (second panel from top), the β-loop (second panel from bottom) and the β-sheets of the CSD (bottom panel).

**Figure S6: YB-1 prevents SG assembly when overexpressed in cells.**

1. Representative images of HeLa cells overexpressing the indicated proteins and treated with arsenite (200 µM, 1 h). SG assembly is inhibited only upon YB-1 and IGF2BP3 expressions.
2. Statistical analysis of the percentage of cells displaying SGs after the overexpression of indicated proteins and exposure to arsenite. Values are means ± SD for three independent samples.

**Figure S7: YB-1 overexpression prevents SG assembly in HeLa cells treated with hydrogen peroxide and SG assembly in U2OS cells treated with arsenite.**

1. Images of HeLa cells overexpressing indicated HA-tagged YB-1 and treated with arsenite (200 µM, 1 h). The inhibition of SG assembly in cells expressing HA-tagged YB-1 is clearly observed when anti-G3BP-1 and anti-FMRP antibodies were used as SG markers, thus confirming the results obtained by using in situ hybridization with poly(dT) probes to detect SGs (Fig. 6a). Scale bar: 40 µm.
2. Images of HeLa cells overexpressing indicated HA-tagged YB-1 and pretreated with puromycin 2.5 μg/ml 30 min prior to and during hydrogen peroxide (300 µM, 1 h). SGs are smaller than those obtained with arsenite. However, we can clearly observe an inhibition of SG assembly in cells expressing Ha-tagged YB-1. Scale bar: 40 µm.
3. U2OS cells expressing indicated HA-tagged YB-1 were treated with arsenite to generate SGs. he total area of SGs per cell was measured as explained in b). **, p < 0.01; t-test with two tails. Scale bar: 40 µm.

**Figure S8: RNA-unwinding defective YB-1 prevents SG assembly when overexpressed in cells.**

1. Anti-YB-1 and anti-HA antibodies were used to quantify the expression level of total YB-1 and HA-tagged YB-1 in HeLa cells expressing HA-tagged YB-1 mutants after a pretreatment with siRNA targeting the 5’UTR of YB-1. Then, an analysis at the single cell level indicates the expression levels of total YB-1 and Ha-Tagged YB-1. The scatter plot shows the increase in total YB-1 expression versus the expression of exogenous HA-tagged YB-1. The results of a linear regression (y=ax+b) are given for each condition.
2. Violin plots representing the total SG area and mean number of SGs per cell in HeLa cells treated with arsenite (200 µM, 1h), after the addback of indicated YB-1 mutants (Duplicate of Fig. 7e).

**Figure S9: Large scale images of SG disassembly in YB-1-poor or -rich HeLa cells.**

1. Controls showing the decrease in YB-1 level after indicated siRNA treatments.
2. Hela cells treated with indicated siRNAs, were exposed to arsenite (1h, 200 µM). Arsenite was then washed out to initiate SG disassembly.

**Figure S10:** Wild type YB-1 added back to siRNA-treated cells increases mRNA translation to a larger extent than RNA-unwinding defective mutant.

1. Translation level was assessed at the single cell level in HeLa cells expressing HA-tagged YB-1 versus HA-YB-1 expression (anti-HA). Hela cells have been pretreated with siRNA to decrease endogenous YB-1 expression by targeting the 3’UTR of YB-1. The slope was measured by using a linear regression. The results were given with a 95% confidence bounds. We controlled that the level of mRNA remained unchanged after YB-1 overexpression (data not shown). **, p < 0.05; t-test with two tails.
