# Supplementary material for: YB-1 unwinds mRNA secondary structures in vitro and negatively regulates stress granule assembly in HeLa cells
Source: Nucleic Acids Res. 2021 Sep 1;49(17):10061–81. doi: 10.1093/nar/gkab748 (PMC8464072; doi:10.1093/nar/gkab748)
Supplement: gkab748_Supplemental_Files [file gkab748_supplemental_files.zip › Table 2.docx]

**Table 2.** Hydrogen bonds occupancy (%) for arginine and lysine residues of CSD loop 3 and CTD in interaction with the RNA stem during 200 ns of MD simulation, for the WT, R97A/K98A and K137A/Y138A complexes. Hydrogen (H) bonds were counted between donors (D) and acceptors (A) provided that the D-A distance is less than 3.0 A and the D-H-A angle is less than 20 degrees.

| **H-bond occupancy (%)** | | **WT** | **R97A-K98A** | **K137A-Y138A** |  | **H-bond occupancy (%)** | | **WT** | **R97A-K98A** | **K137A-Y138A** |
| --- | --- | --- | --- | --- | --- | --- | --- | --- | --- | --- |
| **RNA base pairs** | **Residues** |  |  |  |  | **RNA base pairs** | **Residues** |  |  |  |
| **A1** | **R152** |  |  | 1.2 |  | **C6** | **R97** | 0.2 |  | 4.69 |
|  | **R156** | 0.1 |  |  |  |  | **K98** | 1.9 |  |  |
| **U24** | **R156** |  |  | 0.65 |  |  | **R142** | 0.1 |  |  |
|  | **Total** | 0.1 | 0 | 1.85 |  | **G19** | **R147** | 1.75 |  |  |
| **C2** | **R142** |  | 0.15 | 0.15 |  |  | **R150** |  |  | 0.05 |
|  | **R150** |  |  | 1.64 |  |  | **R151** | 1.9 |  | 1.5 |
|  | **R151** | 15.84 |  |  |  |  | **Total** | 5.85 | 0 | 6.24 |
|  | **R152** | 9.15 |  | 6.08 |  | **A7** | **R97** | 3.9 |  | 12.99 |
|  | **R156** | 0.5 |  |  |  |  | **K98** | 9.99 |  |  |
| **G23** | **R156** |  |  | 3.59 |  | **U18** | **R147** | 1 |  |  |
|  | **Total** | 25.49 | 0.15 | 11.46 |  |  | **R151** |  |  | 16.23 |
| **A3** | **K98** |  |  | 0.6 |  |  | **R156** |  | 1.7 |  |
|  | **R142** |  | 0.2 |  |  |  | **Total** | 14.89 | 1.7 | 29.22 |
|  | **R150** | 1.75 |  | 2.8 |  | **G8** | **R97** | 24.83 |  | 11.54 |
|  | **R151** | 9.14 |  |  |  | **C17** | **R97** |  |  | 1.15 |
|  | **R152** | 0.5 |  | 13.19 |  |  | **R147** | 10.84 |  |  |
| **U22** | **R156** |  |  | 2 |  |  | **R151** |  | 16.98 | 1.65 |
|  | **Total** | 11.39 | 0.2 | 18.59 |  |  | **R152** |  | 1.1 |  |
| **G4** | **K98** |  |  | 2.7 |  |  | **R156** |  | 2.4 |  |
|  | **R142** | 1.2 |  |  |  |  | **Total** | 35.67 | 20.48 | 14.34 |
|  | **R147** | 0.2 |  |  |  | **A9** | **R97** | 27.12 |  | 0.25 |
|  | **R150** | 16.53 |  |  |  | **U16** | **R97** | 0.05 |  |  |
|  | **R151** | 11.94 |  |  |  |  | **R147** | 7.69 | 0.3 | 0.05 |
|  | **R152** |  |  | 11.14 |  |  | **R151** |  | 0.1 |  |
| **C21** | **R156** |  |  | 2.4 |  |  | **R156** |  | 8.89 |  |
|  | **Total** | 29.87 | 0 | 16.24 |  |  | **Total** | 34.86 | 9.29 | 0.3 |
| **A5** | **R97** |  |  | 3.1 |  |  |  |  |  |  |
|  | **K98** |  |  | 0.8 |  |  |  |  |  |  |
|  | **R142** | 38.16 |  |  |  |  |  |  |  |  |
|  | **R150** | 0.15 |  |  |  |  |  |  |  |  |
|  | **R151** | 0.2 |  |  |  |  |  |  |  |  |
|  | **R152** |  |  | 0.05 |  |  |  |  |  |  |
| **U20** | **R147** | 0.45 |  |  |  |  |  |  |  |  |
|  | **R151** | 2.85 |  |  |  |  |  |  |  |  |
|  | **R152** |  |  | 0.55 |  |  |  |  |  |  |
|  | **R156** |  |  | 4.25 |  |  |  |  |  |  |
|  | **Total** | 41.81 | 0 | 8.75 |  |  |  |  |  |  |
