# Supplementary material for: YB-1 unwinds mRNA secondary structures in vitro and negatively regulates stress granule assembly in HeLa cells
Source: Nucleic Acids Res. 2021 Sep 1;49(17):10061–81. doi: 10.1093/nar/gkab748 (PMC8464072; doi:10.1093/nar/gkab748)
Supplement: gkab748_Supplemental_Files [file Figures S1 to S10.pdf › Figures S1 to S10.pdf]

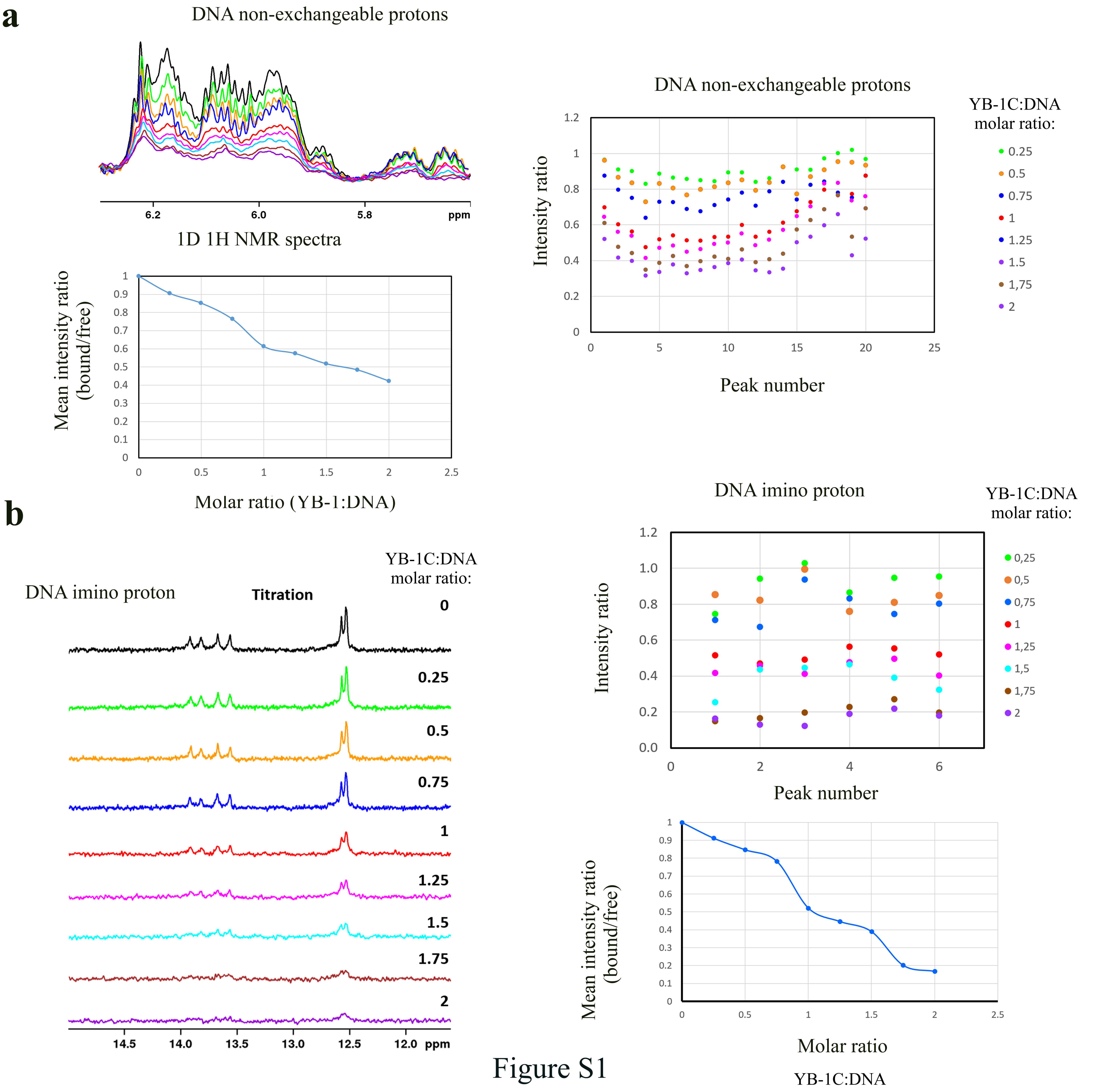
a
DNA non-exchangeable protons
6.2
6.0
5.8
ppm
1D 1H NMR spectra
Mean intensity ratio (bound/free)
Molar ratio (YB-1:DNA)
DNA non-exchangeable protons
Intensity ratio
Peak number
YB-1C:DNA molar ratio:
0.25
0.5
0.75
1
1.25
1.5
1,75
2
b
DNA imino proton
Titration
YB-1C:DNA molar ratio:
0
0.25
0.5
0.75
1
1.25
1.5
1.75
2
14.5
14.0
13.5
13.0
12.5
12.0
ppm
DNA imino proton
Intensity ratio
Peak number
YB-1C:DNA molar ratio:
0,25
0,5
0,75
1
1,25
1,5
1,75
2
Mean intensity ratio (bound/free)
Molar ratio
YB-1C:DNA

Figure S1

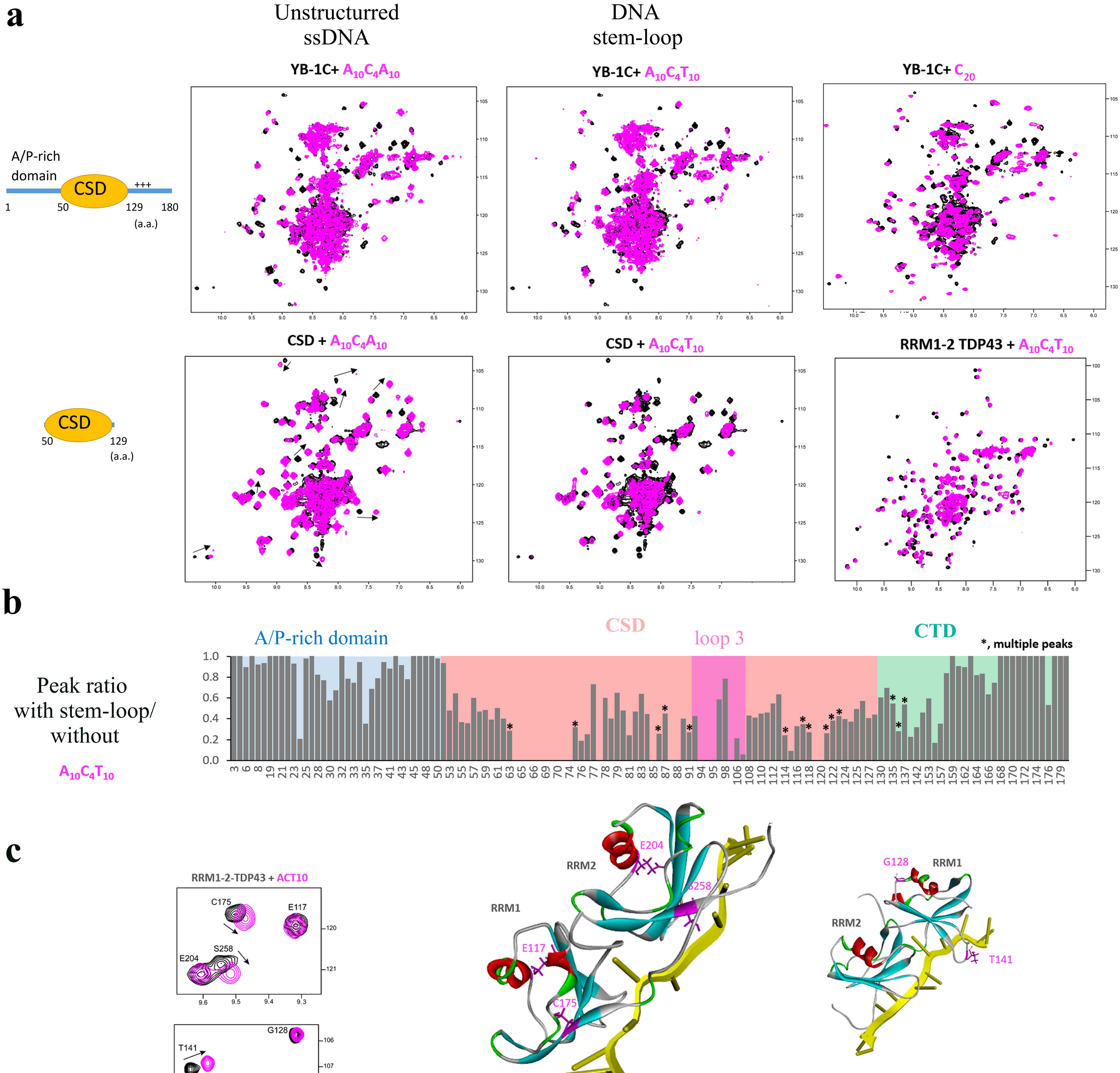
a
Unstructurred ssDNA
DNA stem-loop
YB-1C+ $A_{10}C_4A_{10}$
YB-1C+ $A_{10}C_4T_{10}$
YB-1C+ C_{20}
A/P-rich domain
CSD
+++
1
50
129
180
(a.a.)
CSD + $A_{10}C_4A_{10}$
CSD + $A_{10}C_4T_{10}$
RRM1-2 TDP43 + $A_{10}C_4T_{10}$
b
A/P-rich domain
CSD
loop 3
CTD
*, multiple peaks
Peak ratio with stem-loop/ without
$A_{10}C_4T_{10}$
c
RRM1-2-TDP43 + ACT10
C175
E117
S258
E204
T141
G128
RRM2
RRM1
E204
S258
E117
C175
G128
RRM1
RRM2
T141

Figure S2

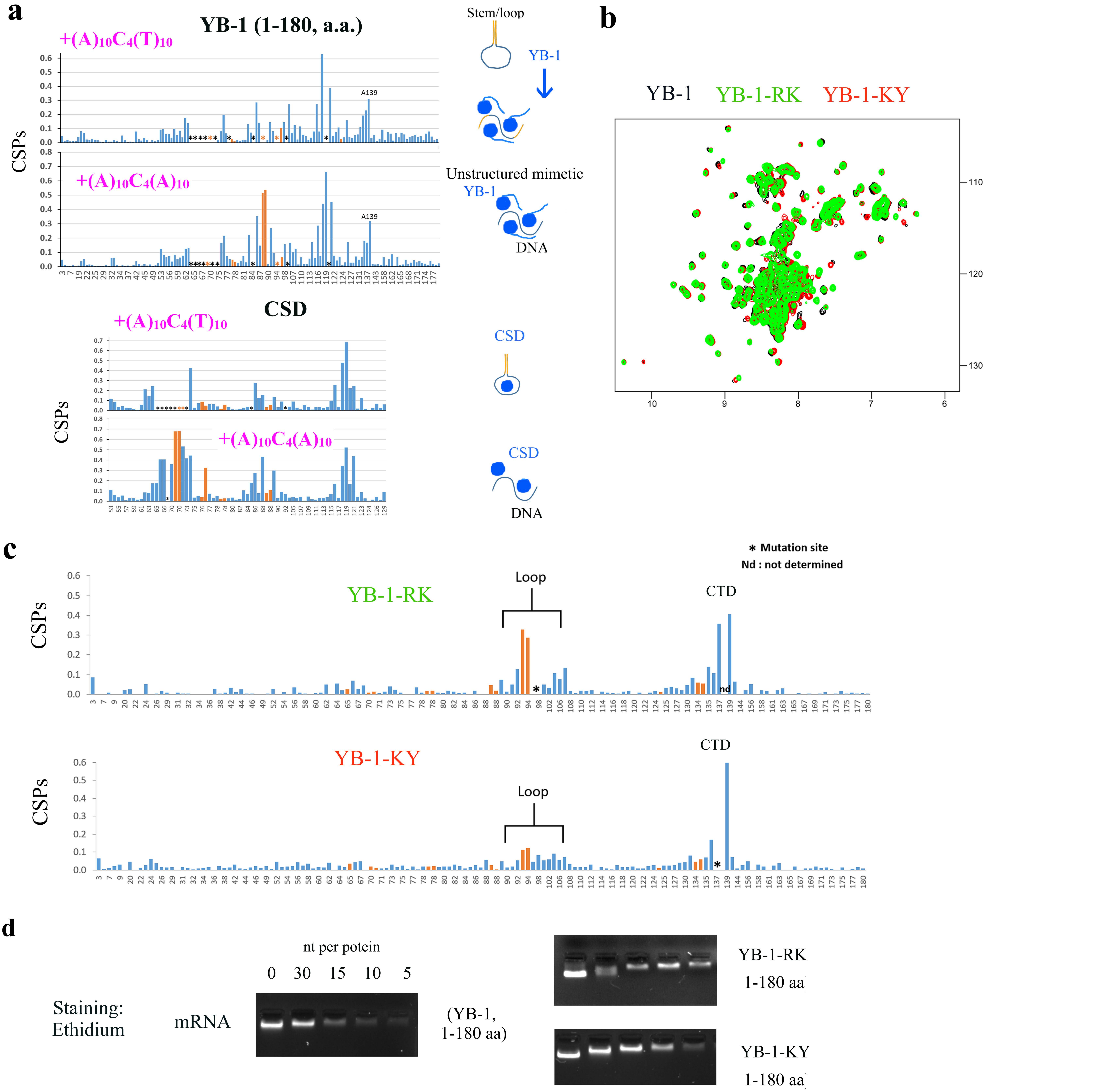
a
YB-1 (1-180, a.a.)
+(A)10C4(T)10
+(A)10C4(A)10
CSPs
A139
Stem/loop
YB-1
Unstructured mimetic
YB-1
DNA
CSD
+(A)10C4(T)10
+(A)10C4(A)10
CSPs
CSD
CSD
DNA
b
YB-1
YB-1-RK
YB-1-KY
c
* Mutation site
Nd : not determined
YB-1-RK
Loop
CTD
CSPs
nd
YB-1-KY
Loop
CTD
CSPs
d
nt per potein
0 30 15 10 5
Staining: Ethidium
mRNA
(YB-1, 1-180 aa)
YB-1-RK 1-180 aa
YB-1-KY 1-180 aa

Figure S3

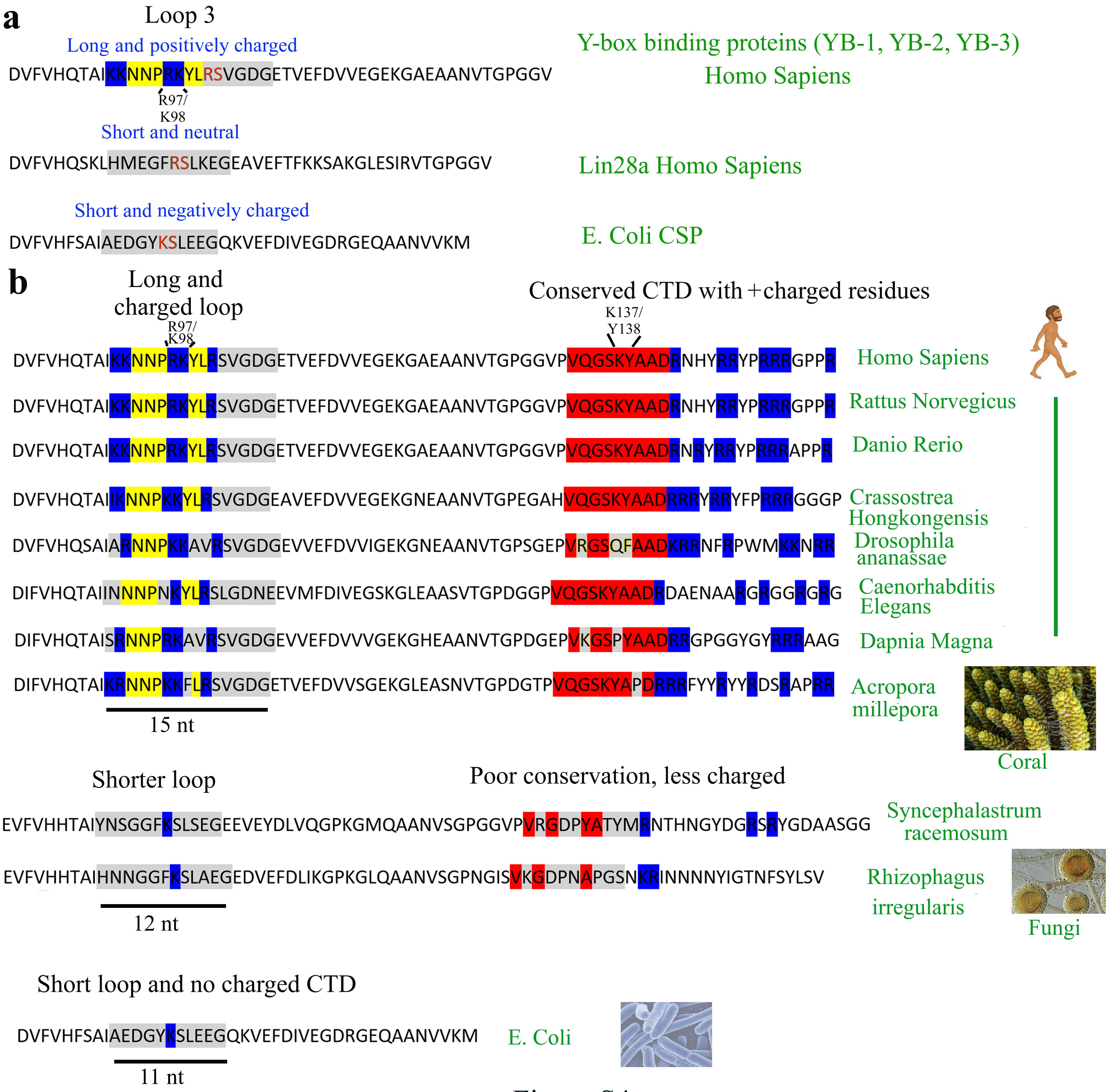
a
Loop 3
Long and positively charged
DVFVHQTAIKKNNPRKYLRSVGDGETVEFDVVEGEKGAEAANVTGPGGV
R97/
K98
Y-box binding proteins (YB-1, YB-2, YB-3)
Homo Sapiens
Short and neutral
DVFVHQSKLHMEGFRSLKEGEAVEFTFKKSAKGLESIRVTGPGGV
Lin28a Homo Sapiens
Short and negatively charged
DVFVHFSAIAEDGYKSLEEGQKVEFDIVEGDRGEQAANVVKM
E. Coli CSP
b
Long and charged loop
R97/
K98
Conserved CTD with +charged residues
K137/
Y138
DVFVHQTAIKKNNPRKYLRSVGDGETVEFDVVEGEKGAEAANVTGPGGVPVQGSKYAADRNHYRRYPRRRGPPR
Homo Sapiens
DVFVHQTAIKKNNPRKYLRSVGDGETVEFDVVEGEKGAEAANVTGPGGVPVQGSKYAADRNHYRRYPRRRGPPR
Rattus Norvegicus
DVFVHQTAIKKNNPRKYLRSVGDGETVEFDVVEGEKGAEAANVTGPGGVPVQGSKYAADRNRYRRYPRRRAPPR
Danio Rerio
DVFVHQTAIKNNPKKYLRSVGDGEAVEFDVVEGEKGNEAANVTGPEGAHVQGSKYAADRRRYRRYFPRRRGGGP
Crassostrea Hongkongensis
DVFVHQSAIARNNPKKAVRSVGDGEVVEFDVVIGEKGNEAANVTGPSGEPVRGSQFAADKRRNFRPWMKKNRR
Drosophila ananassae
DIFVHQTAIINNNPNKYLRSLGDNEEVMFDIVEGSKGLEAASVTGPDGGPVQGSKYAADRDAENAARGRGGRGRG
Caenorhabditis Elegans
DIFVHQTAISRNNPRKAVRSVGDGEVVEFDVVVGEKGHEAANVTGPDGEPVKGSPYAADRRGPGGYGYRRRAAG
Dapnia Magna
DIFVHQTAIKRNNPKKFLRSVGDGETVEFDVVSGEKGLEASNVTGPDGTPVQGSKYAPDRRRFYYRYYRDSRAPRR
Acropora millepora
15 nt
Coral
Shorter loop
Poor conservation, less charged
EVFVHHTAIYNSGGFKSLSEGEEVEYDLVQGPKGMQAANVSGPGGVPVRGDPYATYMRNTHNGYDGRSRYGDAASGG
Syncephalastrum racemosum
EVFVHHTAIHNNGGFKSLAEGEDVEFDLIKGPKGLQAANVSGPNGISVKGDPNAPGSNKRINNNNYIGTNFSYLSV
Rhizophagus irregularis
12 nt
Fungi
Short loop and no charged CTD
DVFVHFSAIAEDGYKSLEEGQKVEFDIVEGDRGEQAANVVKM
E. Coli
11 nt

Figure S4

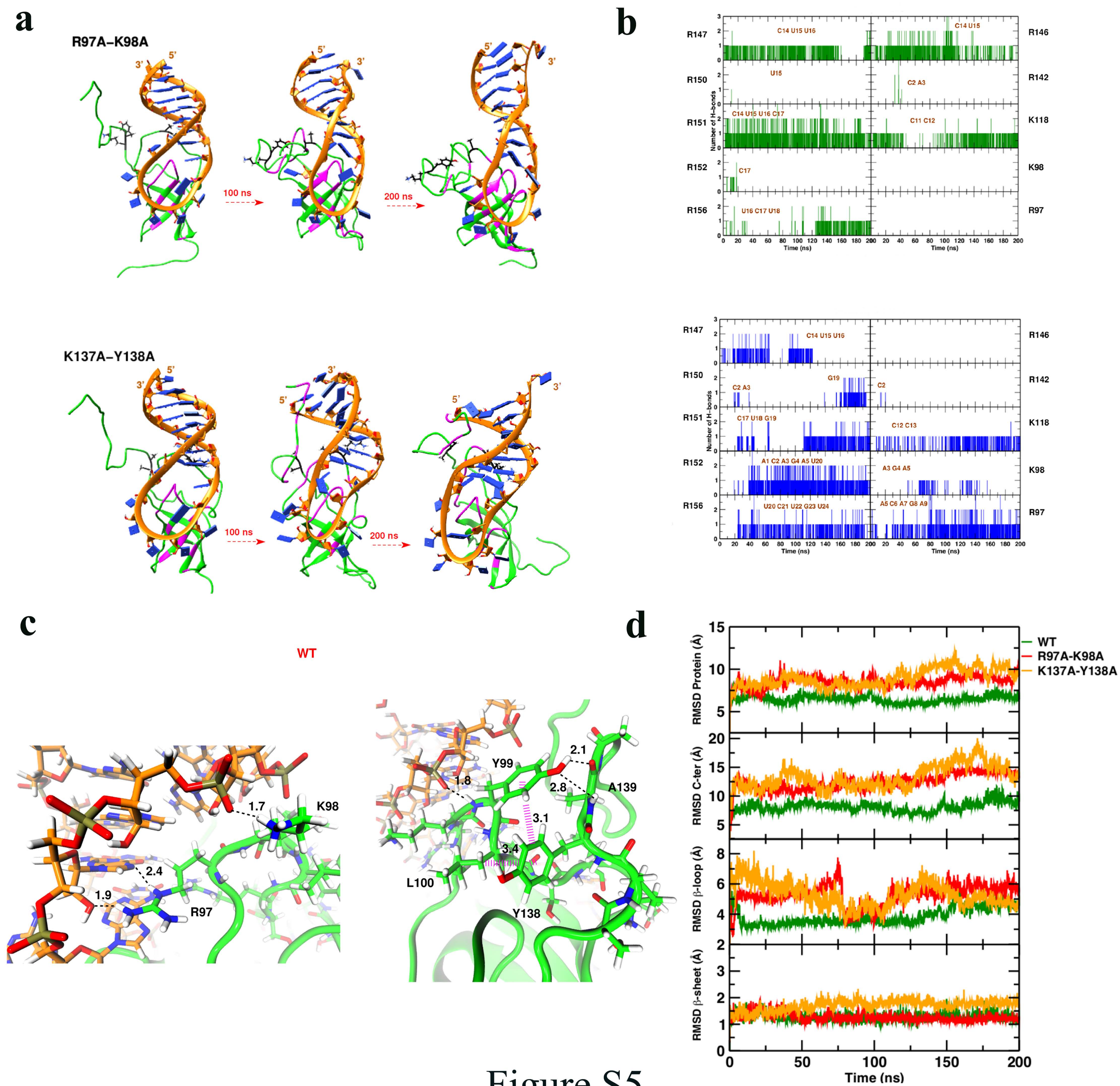
a
R97A–K98A
100 ns
200 ns
K137A–Y138A
b
Number of H-bonds
Time (ns)
R147
R150
R151
R152
R156
R146
R142
K118
K98
R97
C14 U15 U16
C14 U15
U15
C2 A3
C14 U15 U16 C17
C11 C12
C17
U16 C17 U18
G19
C2
C17 U18 G19
C12 C13
A1 C2 A3 G4 A5 U20
A3 G4 A5
U20 C21 U22 G23 U24
A5 C6 A7 G8 A9
c
WT
K98
R97
Y99
A139
L100
Y138
d
WT
R97A-K98A
K137A-Y138A
RMSD Protein (Å)
RMSD C-ter (Å)
RMSD β-loop (Å)
RMSD β-sheet (Å)
Time (ns)

Figure S5

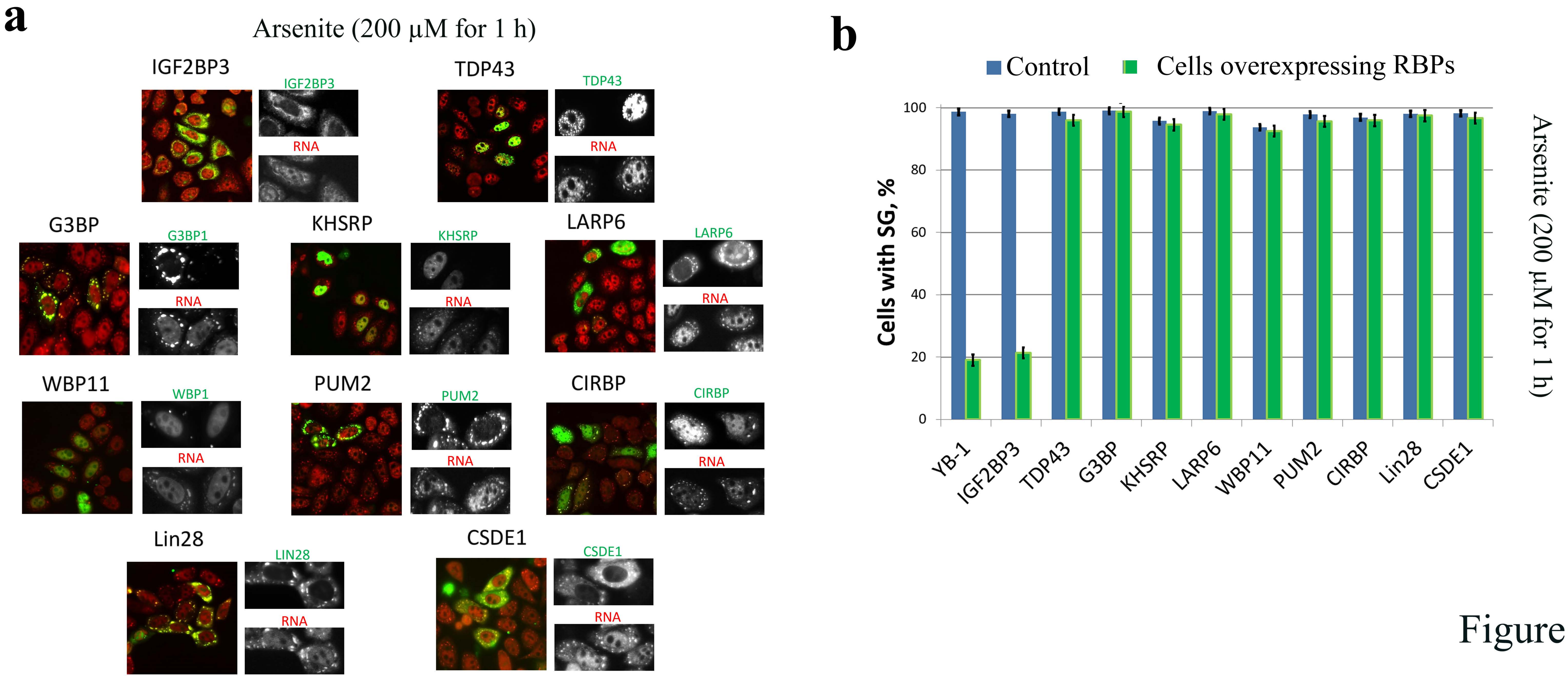
a
Arsenite (200 μM for 1 h)
IGF2BP3
IGF2BP3
RNA
TDP43
TDP43
RNA
G3BP
G3BP1
RNA
KHSRP
KHSRP
RNA
LARP6
LARP6
RNA
WBP11
WBP1
RNA
PUM2
PUM2
RNA
CIRBP
CIRBP
RNA
Lin28
LIN28
RNA
CSDE1
CSDE1
RNA
b
Control
Cells overexpressing RBPs
Cells with SG, %
100
80
60
40
20
0
YB-1
IGF2BP3
TDP43
G3BP
KHSRP
LARP6
WBP11
PUM2
CIRBP
Lin28
CSDE1
Arsenite (200 μM for 1 h)

Figure S6

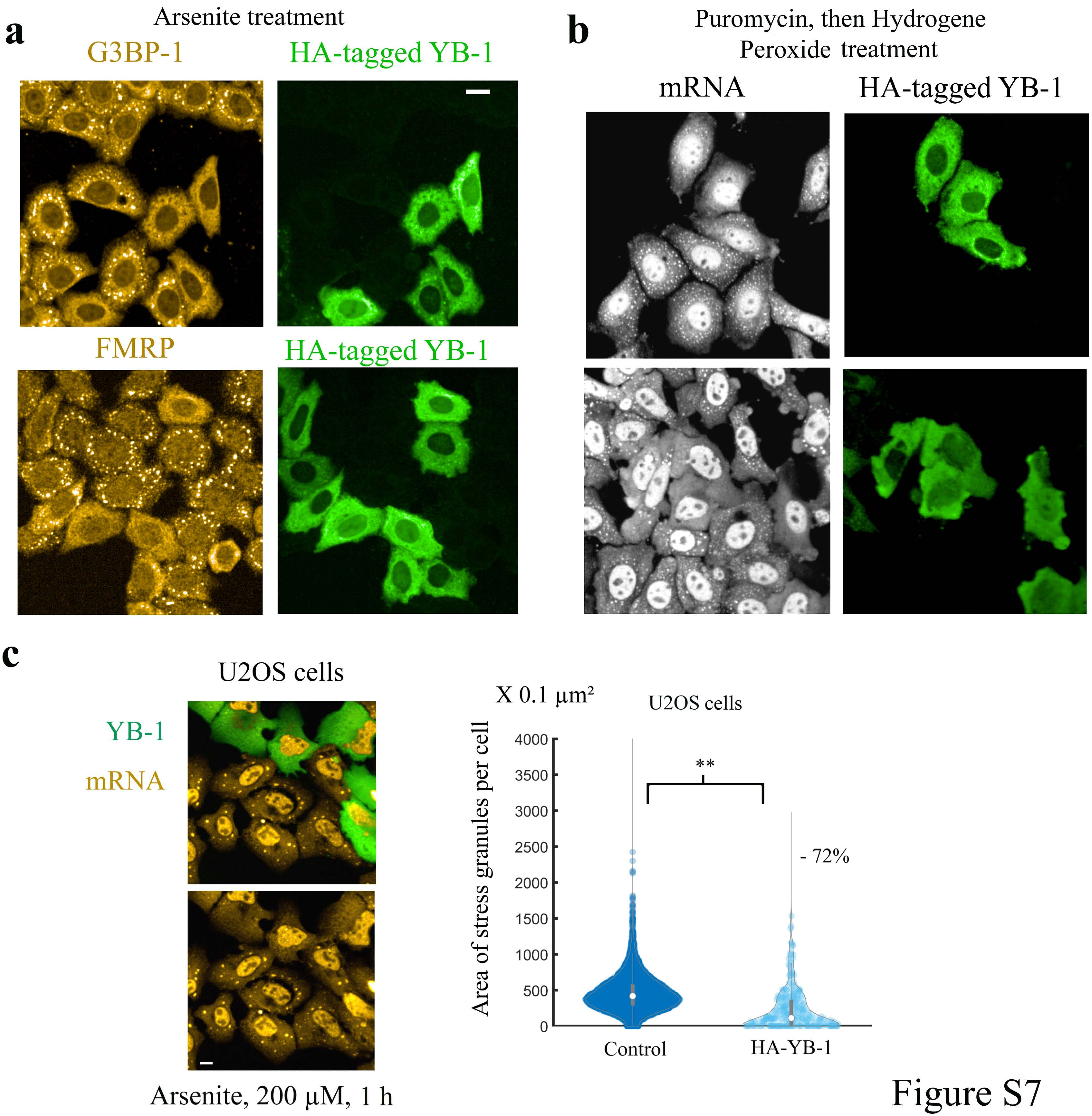
a
Arsenite treatment
G3BP-1
HA-tagged YB-1
FMRP
HA-tagged YB-1
b
Puromycin, then Hydrogene
Peroxide treatment
mRNA
HA-tagged YB-1
c
U2OS cells
YB-1
mRNA
Arsenite, 200 μM, 1 h
X 0.1 μm²
U2OS cells
Area of stress granules per cell
4000
3500
3000
2500
2000
1500
1000
500
0
**
- 72%
Control
HA-YB-1

Figure S7

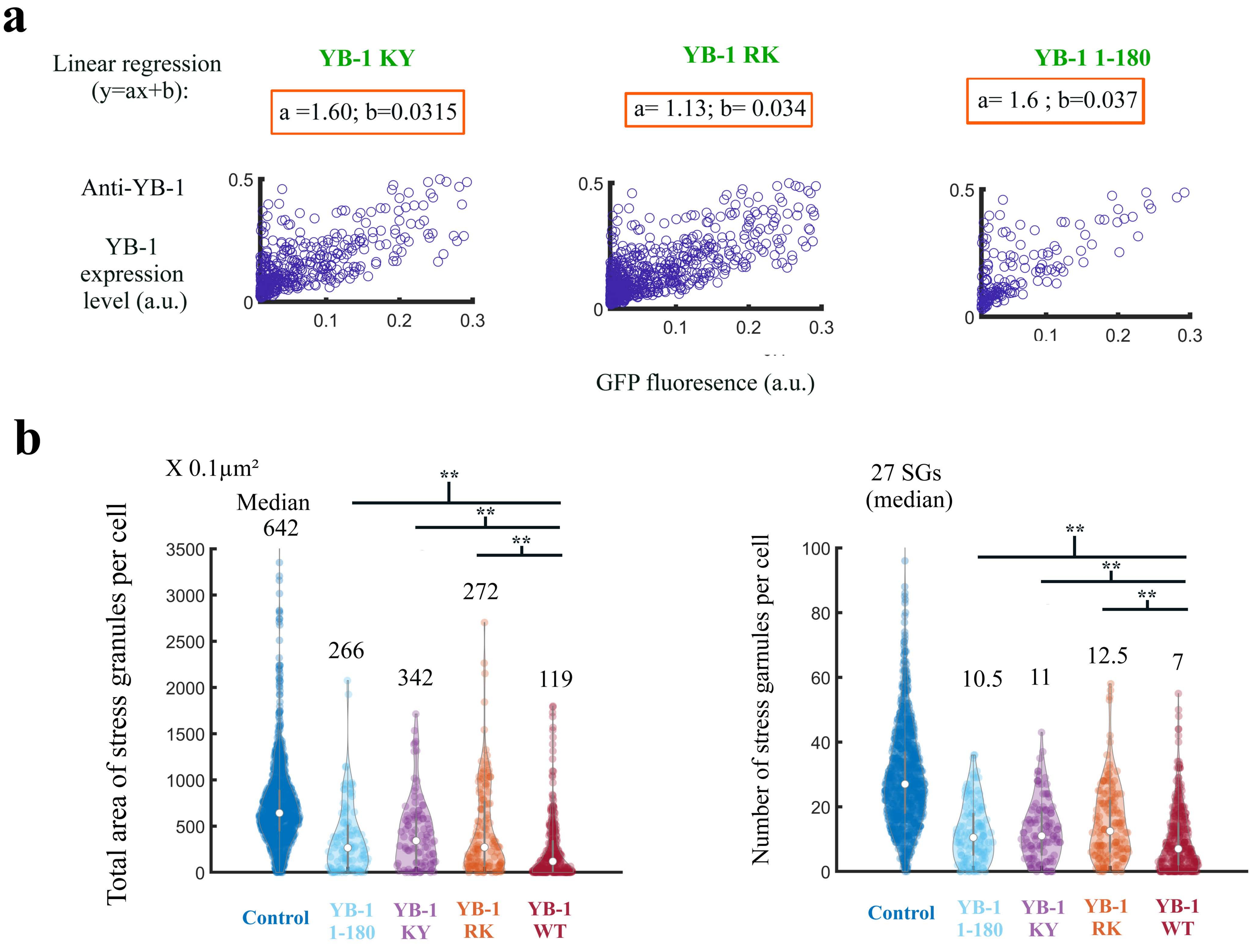
a
Linear regression (y=ax+b):
YB-1 KY
a =1.60; b=0.0315
YB-1 RK
a= 1.13; b= 0.034
YB-1 1-180
a= 1.6 ; b=0.037
Anti-YB-1
YB-1 expression level (a.u.)
GFP fluoresence (a.u.)
b
X 0.1μm²
Median 642
266
342
272
119
Total area of stress granules per cell
27 SGs (median)
10.5
11
12.5
7
Number of stress garnules per cell
Control
YB-1 1-180
YB-1 KY
YB-1 RK
YB-1 WT

Figure S8

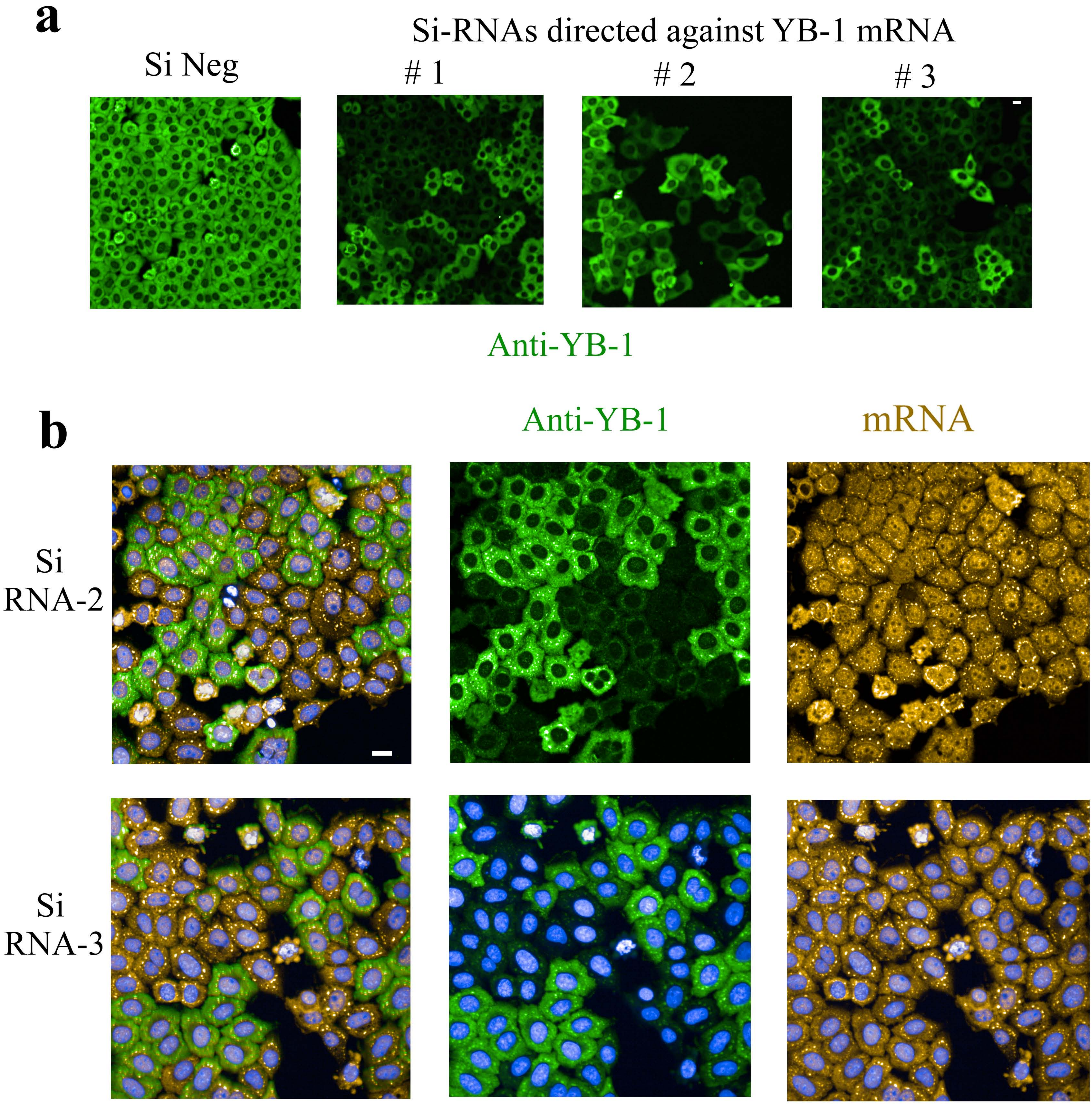
a
Si-RNAs directed against YB-1 mRNA
Si Neg
1
2
3
Anti-YB-1
b
Anti-YB-1
mRNA
Si RNA-2
Si RNA-3

Figure S9

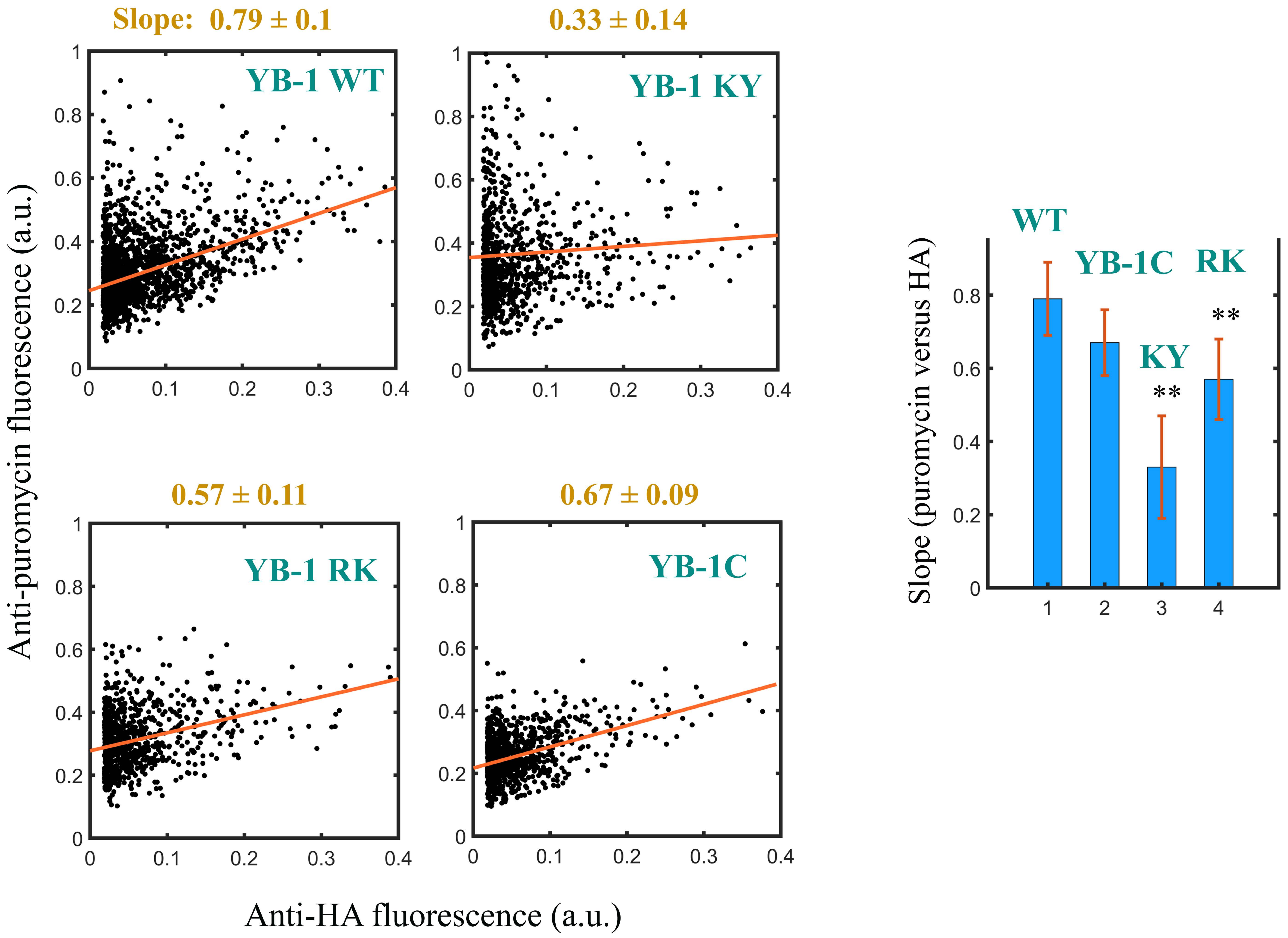

Slope: 0.79 ± 0.1
YB-1 WT
0.33 ± 0.14
YB-1 KY
0.57 ± 0.11
YB-1 RK
0.67 ± 0.09
YB-1C
Anti-puromycin fluorescence (a.u.)
Anti-HA fluorescence (a.u.)
Slope (puromycin versus HA)
WT
YB-1C
RK
KY
**
**

Figure S10
